# Genetic Studies of Hypertrophic Cardiomyopathy in Singaporeans Identify Variants in *TNNI3* and *TNNT2* That Are Common in Chinese Patients

**DOI:** 10.1161/CIRCGEN.119.002823

**Published:** 2020-08-20

**Authors:** Chee Jian Pua, Nevin Tham, Calvin W.L. Chin, Roddy Walsh, Chiea Chuen Khor, Christopher N. Toepfer, Giuliana G. Repetti, Amanda C. Garfinkel, Jourdan K. Ewoldt, Paige Cloonan, Christopher S. Chen, Shi Qi Lim, Jiashen Cai, Li Yang Loo, Siew Ching Kong, Charleston W.K. Chiang, Nicola Whiffin, Antonio de Marvao, Pei Min Lio, An An Hii, Cheng Xi Yang, Thu Thao Le, Yasmin Bylstra, Weng Khong Lim, Jing Xian Teo, Kallyandra Padilha, Gabriela V. Silva, Bangfen Pan, Risha Govind, Rachel J. Buchan, Paul J.R. Barton, Patrick Tan, Roger Foo, James W.L. Yip, Raymond C.C. Wong, Wan Xian Chan, Alexandre C. Pereira, Hak Chiaw Tang, Saumya Shekhar Jamuar, James S. Ware, Jonathan G. Seidman, Christine E. Seidman, Stuart A. Cook

**Affiliations:** National Heart Centre Singapore (C.J.P., N.T., C.W.L.C., S.Q.L., S.C.K., P.M.L., A.A.H., C.X.Y., T.T.L., H.C.T., S.A.C.).; Yong Loo Lin School of Medicine, National University Singapore (C.J.P., L.Y.L.).; Duke-National University of Singapore Medical School (C.W.L.C., J.C., S.S.J., S.A.C.).; Department of Clinical and Experimental Cardiology, Heart Center, Amsterdam Cardiovascular Sciences, Amsterdam UMC, University of Amsterdam, the Netherlands (R.W.).; Genome Institute of Singapore (C.C.K., P.T., R.F.).; Department of Genetics, Harvard Medical School, Boston, MA (C.N.T., G.G.R., A.C.G., K.P., G.V.S., A.C.P., J.G.S., C.E.S.).; Radcliffe Department of Medicine, University of Oxford, United Kingdom (C.N.T.).; Department of Biomedical Engineering, Boston University, MA (J.K.E., P.C., C.S.C.).; Center for Genetic Epidemiology, University of Southern California (C.W.K.C.).; Center for Neurobehavioral Genetics, University of California, Los Angeles (C.W.K.C.).; Cardiovascular Research Center, Royal Brompton Hospital, London, United Kingdom (N.W., A.d.M., R.G., R.J.B., P.J.R.B., J.S.W., S.A.C.).; National Heart and Lung Institute, Imperial College London, United Kingdom (N.W., A.d.M., R.G., R.J.B., P.J.R.B., J.S.W., S.A.C.).; SingHealth/Duke-NUS Precision Medicine Inst, Singapore (Y.B., W.K.L., J.X.T., P.T., S.S.J.).; Laboratory of Genetics and Molecular Cardiology, Heart Institute (InCor)-University of São Paulo Medical School, Brazil (K.P., G.V.S., A.C.P.).; Cardiovascular Research Institute, National University Health System, Singapore (B.P., R.F.).; Cardiology Department, National University Heart Centre, Singapore (J.W.L.Y., R.C.C.W., W.X.C.).; KK Women’s and Children’s Hospital, Singapore (S.S.J.).; SingHealth Duke-NUS Genomic Medicine Centre, Singapore (S.S.J.).; Cardiovascular Division, Brigham and Women’s Hospital, Howard Hughes Medical Institute, Boston, MA (C.E.S.).

**Keywords:** cardiomyopathies, hypertrophy, population, troponin I, troponin T

## Abstract

**Background:**

To assess the genetic architecture of hypertrophic cardiomyopathy (HCM) in patients of predominantly Chinese ancestry.

**Methods:**

We sequenced HCM disease genes in Singaporean patients (n=224) and Singaporean controls (n=3634), compared findings with additional populations and White HCM cohorts (n=6179), and performed in vitro functional studies.

**Results:**

Singaporean HCM patients had significantly fewer confidently interpreted HCM disease variants (pathogenic/likely pathogenic: 18%, *P*<0.0001) but an excess of variants of uncertain significance (24%, *P*<0.0001), as compared to Whites (pathogenic/likely pathogenic: 31%, excess of variants of uncertain significance: 7%). Two missense variants in thin filament encoding genes were commonly seen in Singaporean HCM (TNNI3:p.R79C, disease allele frequency [AF]=0.018; TNNT2:p.R286H, disease AF=0.022) and are enriched in Singaporean HCM when compared with Asian controls (TNNI3:p.R79C, Singaporean controls AF=0.0055, *P*=0.0057, genome aggregation database-East Asian AF=0.0062, *P*=0.0086; TNNT2:p.R286H, Singaporean controls AF=0.0017, *P*<0.0001, genome aggregation database-East Asian AF=0.0009, *P*<0.0001). Both these variants have conflicting annotations in ClinVar and are of low penetrance (TNNI3:p.R79C, 0.7%; TNNT2:p.R286H, 2.7%) but are predicted to be deleterious by computational tools. In population controls, TNNI3:p.R79C carriers had significantly thicker left ventricular walls compared with noncarriers while its etiological fraction is limited (0.70 [95% CI, 0.35–0.86]) and thus TNNI3:p.R79C is considered variant of uncertain significance. Mutant TNNT2:p.R286H iPSC-CMs (induced pluripotent stem cells derived cardiomyocytes) show hypercontractility, increased metabolic requirements, and cellular hypertrophy and the etiological fraction (0.93 [95% CI, 0.83–0.97]) support the likely pathogenicity of TNNT2:p.R286H.

**Conclusions:**

As compared with Whites, Chinese HCM patients commonly have low penetrance risk alleles in *TNNT2* or *TNNI3* but exhibit few clinically actionable HCM variants overall. This highlights the need for greater study of HCM genetics in non-White populations.

## Abstract 2

Supplemental Digital Content is available in the text.

Hypertrophic cardiomyopathy (HCM) is a common Mendelian disease with prevalence of up to 1 in 500 people^1^ and is diagnosed by the presence of left ventricular hypertrophy that cannot be explained by systemic or other cardiac diseases.^2^ HCM is generally thought of as an autosomal dominant Mendelian disease of variable penetrance and expressivity where disease-causing variants, mostly with allele frequencies (AF) <0.0001, are found in up to 50% of patients.^3^ *MYH7* was the first HCM gene identified^4^ and rare variants are observed in ≈13% of all HCM cases in one of the largest published cohorts (n=6179) of patients with HCM.^3^ *MYBPC3* is the most prevalent HCM-associated gene and seen in ≈17% cases, while other sarcomere-encoding genes (*ACTC1, MYL2, MYL3, TNNI3, TNNT2*, and *TPM1*) account for most of the remaining cases with a genetic causation. Although >30 other genes are often included in HCM genetic tests,^5^ recent evaluation of these genes showed limited or no evidence of disease association for most of these genes.^6^

The variant classification framework of the American College of Medical Genetics and Genomics and the Association for Molecular Pathology (ACMG/AMP) provides an evidence-based approach to classify the pathogenicity of sequence variants.^7^ The release of publicly available Genome Aggregation Database (gnomAD) provides a reference population to refine our understanding of the spectrum of rare variation in the human genome and to rule out alleles that are insufficiently rare to be causative of penetrant Mendelian disease.^8,9^ However, this approach needs to be used with caution as variants that are not very rare, including founder and recurrent variants, have been reported in HCM.^10^ Previous studies identified 2 founder variants of *MYBPC3* that are significantly enriched in HCM cases in South Asian^11^ and Icelandic populations,^12^ which are now mostly classified as pathogenic by clinical genetics laboratories in ClinVar despite being relatively common in specific populations. It is important to have a firm understanding of the genetic architecture of HCM in a given population, and to recognize population-specific alleles, including founder variants, before the ACMG/AMP framework can be confidently applied in that population.^7^

In this study, we assessed the genetic architecture of HCM in Singapore in unrelated patients of predominantly Chinese ancestry. Variant calling and comparison with other HCM and control cohorts and the subsequent analyses of variants enriched in Singaporean HCM cases are outlined in a study overview cartoon in Figure 1.

**Figure 1.**
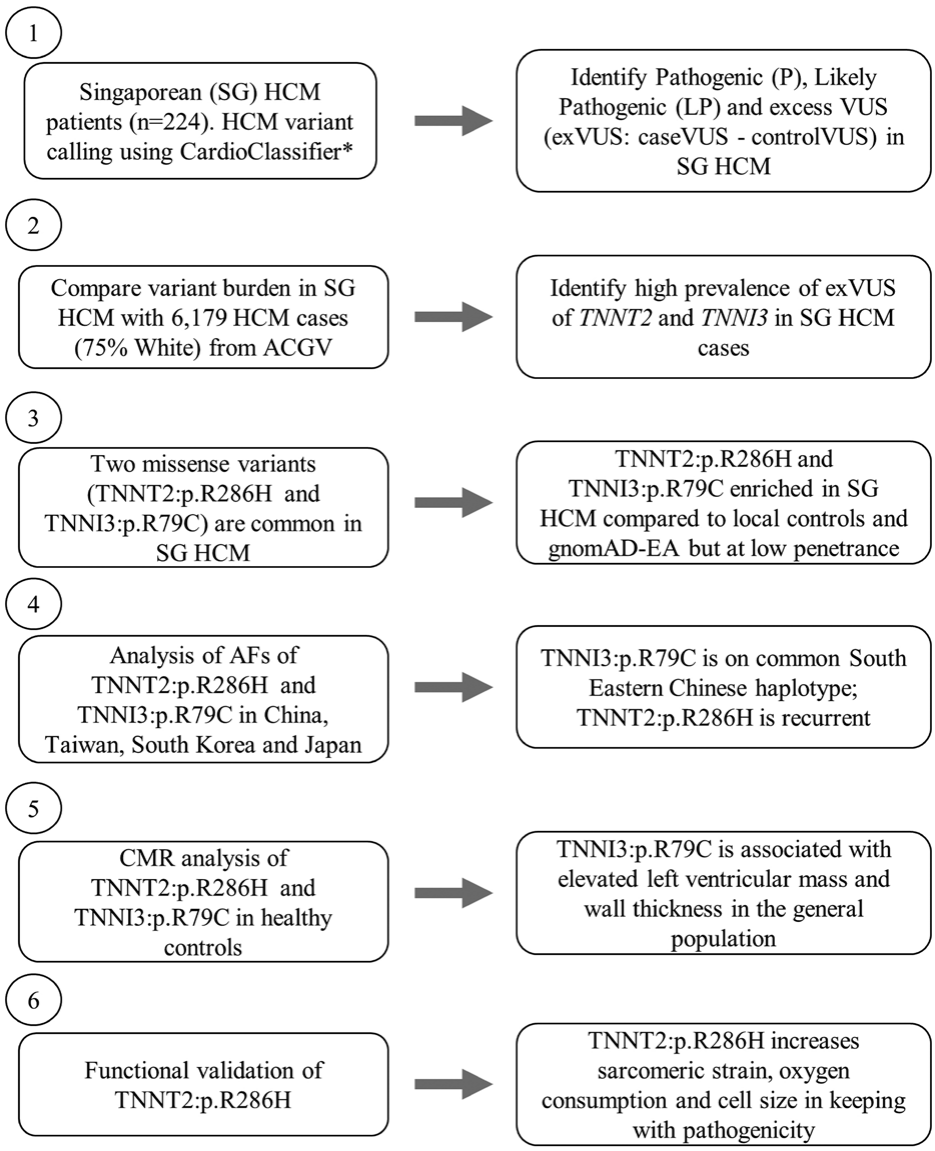
**Overview of the study design outlining the major components of the study and the various disease and control populations used.** ACGV indicates atlas of cardiac genetic variation^3,13,14^; AF, allele frequency; CMR, cardiac magnetic resonance; EA, East Asian; exVUS, excess variant of uncertain significance; HCM, hypertrophic cardiomyopathy; LP, likely pathogenic; P, pathogenic; and SG, Singaporean. *https://www.cardioclassifier.org/15

## Methods

We prospectively recruited unrelated Singaporean patients with HCM (n=224) and Singaporean controls (n=3634)^13^ and performed targeted resequencing.^14^ Variants of 15 genes^6^ either robustly associated with HCM or well-validated pheno/genocopies (*ACTC1, CSRP3, FHL1, GLA, LAMP2, MYBPC3, MYH7, MYL2, MYL3, PLN, PRKAG2, TNNC1, TNNI3, TNNT2*, and *TPM1*) were evaluated,^15^ and we compared findings with reference population data sets,^9,16–18^ White HCM cohorts (n=6179)^3,19,20^ and performed functional studies using iPSC-CMs (induced pluripotent stem cells derived cardiomyocytes).^21,22^ Detailed methods are available in the Data Supplement. All Singaporean participants gave written informed consent to participate in this ethics board-approved study, which was performed in accordance with local Tissue Acts. Supporting data are available either within the article and in the Data Supplement or will be available on a reasonable request to the corresponding author due to privacy issue and national laws under the provision that data may not leave the hospital/center premises.

## Results

### The Genetic Architecture of HCM in Singapore

We sequenced 15 genes robustly associated with HCM and HCM pheno/genocopies in 224 unrelated, predominantly self-reported Chinese (78%) patients with a diagnosis of HCM. Preliminary annotations of variant pathogenicity were determined using CardioClassifier^15^ with subsequent curation and validation by a clinical geneticist according to ACMG/AMP guidelines (Tables II and III in the Data Supplement). The estimated contribution of each gene to disease was defined as the excess burden of rare protein-altering variants in cases compared with the population background (case excess), as previously described.^19^ Population stratification assessment was performed using the genotype data of Singaporean HCM and Singaporean control cohorts and principal component analysis, which showed overlapping clusters of cases and controls (Figure I in the Data Supplement).

**Figure 2.**
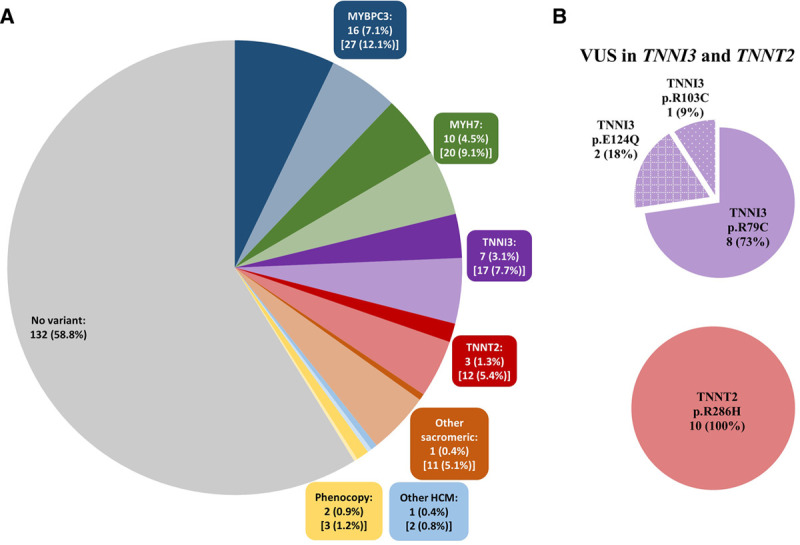
**Pathogenic/likely pathogenic variants and excess variant of uncertain significance (VUS; exVUS = caseVUS%−controlVUS%) in hypertrophic cardiomyopathy (HCM) genes in Singaporean patients with HCM.** **A**, Fifteen genes were assessed including major sarcomeric genes (*MYBPC3*, *MYH7*, *TNNT2*, and *TNNI3*), other sarcomeric genes (*ACTC*, *MYL2*, *MYL3*, *TPM1*, and *TNNC1*), other HCM genes (*CSRP3*, *FHL1*, and *PLN*), and geno/phenocopies (*GLA*, *LAMP*, and *PRKAG2*). The number and percentage refer to the total pathogenic/likely pathogenic (P/LP) case per gene (darker shade) while the number and percentage in parentheses refer to the total case excess of P, LP, and exVUS (lighter shade) as compared to genome aggregation database (gnomAD). **B**, The secondary pie charts show the proportion of all Singaporean HCM patients with TNNI3:p.R79C or TNNT2:p.R286H VUS as compared to other VUS in these genes, depicted overall in (**A**) by lighter shading.

In Singaporean patients with HCM, a significant case excess (*P*<0.0001) of variants was observed in the 2 thick filament genes (*MYBPC3*, 12.1% and *MYH7*, 9.1%) and the 2 main thin filament genes (*TNNI3*, 7.7% and *TNNT2*, 5.4%; Table IV in the Data Supplement). There was insufficient statistical power to study other minor genes in detail, however, the remaining sarcomeric genes (*ACTC1, MYL2, MYL3*, *TNNC1*, and *TPM1*) had a combined case excess of 5.1%. Other HCM genes (*CSRP3, FHL1*, and *PLN*) and pheno/genocopy genes (*GLA*, *LAMP2*, and *PRKAG2*) exhibited few variants (2.0%; Figure 2A and Table IV in the Data Supplement). Over 95% of the variant case excess (>92% of pathogenic/likely pathogenic [P/LP] variants) were observed in the sarcomere-encoding genes. Overall, about 60% of Singaporean patients with HCM had no disease-associated variant in the 15 core HCM genes. The mean left ventricular maximum wall thickness (LVMWT) in Singaporean patients with HCM was 19.2 mm as measured by cardiac magnetic resonance or 20 mm by echocardiogram imaging while the average indexed LV mass was 92.3g/m^2^ (cardiac magnetic resonance). There were no differences in cardiac phenotypes among P/LP variants in sarcomeric-positive carriers and noncarriers (Table V in the Data Supplement).

We then compared the variant case excess frequency in the 15 HCM genes in Singaporeans patients with data from the atlas of cardiac genetic variation comprising up to 6179 HCM cases, mostly (≈75%) White.^3,19,20^ Overall, the excess burden of rare variants (P/LP and variant of uncertain significance [VUS]) in the 15 HCM-associated genes was similar in the 2 cohorts (41% in Singaporean, 38% in White; Table 1). However, Singaporean patients had significantly fewer variants that could be interpreted with sufficient confidence for clinical decision making (P/LP) as compared to the White data set (18% versus 31%, *P*<0.0001), but correspondingly had a much greater excess of VUS (24% versus 7%, *P*<0.0001; Table 1). Interestingly, 2 thin filament genes associated with HCM, but with a modest prevalence of P/LP variants in Whites (case excess: *TNNI3*, 2.0% and *TNNT2*, 1.6%) had a far greater variant case excess in Singaporean HCM (*TNNI3*, 7.7%, *P*<0.0001 and *TNNT2*, 5.4%, *P*=0.0005).

**Table 1.**
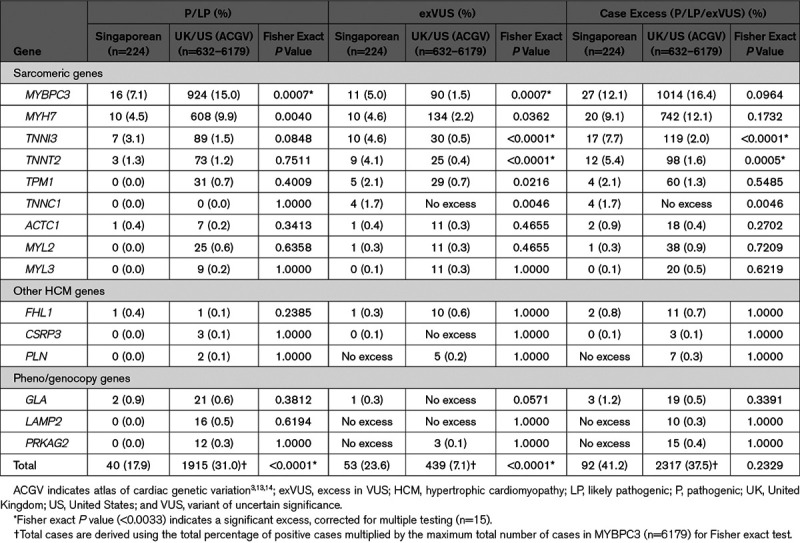
Frequencies of HCM Variant Case Excesses in 15 HCM Genes in Singaporean and White Patients

### TNNI3:p.R79C and TNNT2:p.R286H Variants Are Enriched in Singaporean HCM

We then studied *TNNI3* and *TNNT2* variants in more detail. While there was no significant excess of P/LP variants in these genes (Table 1), 2 distinct VUS, TNNI3:p.R79C (ENST00000344887:c.235C>T) and TNNT2:p.R286H (ENST00000367318:c.857G>A) were commonly observed as heterozygous variant in 8/224 (3.6%) and 10/224 (4.5%) Singaporean patients with HCM, respectively (Figure 2B). Carriers of these variants were self-reported Chinese and were mostly identified as Chinese using principal component analysis (Figure II in the Data Supplement). Singaporean HCM carriers of TNNI3:p. R79C or TNNT2:p.R286H had similar cardiac morphology when compared with HCM patients with P or LP variants in these genes (Table VI in the Data Supplement).

The enrichment of the TNNI3:p.R79C and TNNT2:p.R286H variants in HCM cases compared with White patients was explored further using East Asian controls from gnomAD and also against 3634 local Singaporean controls. TNNT2:p.R286H variants are rare (minor allele frequency [MAF]<0.0001) while TNNI3:p.R79C has a MAF of 0.0004 in gnomAD (Table 2). The AF of TNNI3:p.R79C and TNNT2:p.R286H in gnomAD-East Asian or locally recruited Singaporean controls (in parentheses) was found to be higher: 0.0062 (0.0055) and 0.0009 (0.0017), respectively (Table 2). Similarly, TNNI3:p.R79C is common in Korean (gnomAD v2.1, AF=0.0063) and Japanese (human genetic variation database, AF=0.0041) populations. TNNT2:p.R286H is not reported in either Korean or Japanese subcontinental population control data sets. Both variants are significantly enriched in HCM cases as compared to local Singaporean controls (TNNI3:p.R79C, *P*=0.0057 and TNNT2:p.R286H, *P*<0.0001) and when considered against gnomAD-East Asian (TNNI3:p.R79C, *P*=0.0086 and TNNT2:p.R286H, *P*<0.0001).

**Table 2.**
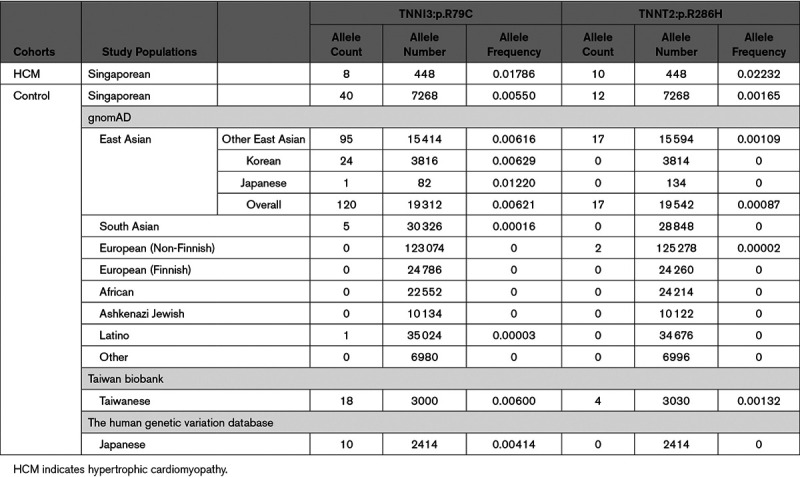
Allele Frequencies of TNNI3:p.R79C (rs3729712) or TNNT2:p.R286H (rs141121678) in Singaporean HCM Cases and Controls and in Different Population Controls

### Attributable Risk in Cases With HCM

We then determined the effect of the 2 thin filament variants on HCM susceptibility. Using Singaporean controls as the population-specific reference, TNNI3:p.R79C has an odds ratio (95% CI) of 3.33 (1.54–7.20) and etiological fraction (95% CI) of 0.70 (0.35–0.86; Table 3). The TNNT2:p.R286H variant has a higher odds ratio (95% CI) of 14.1 (6.03–33.01) and etiological fraction (95% CI) of 0.93 (0.83–0.97). Both TNNI3:p.R79C and TNNT2:p.R286H variants have been reported previously in HCM cases in China and Taiwan^23,24^ but with conflicting assessments of pathogenicity as shown in ClinVar (Table VII in the Data Supplement). TNNI3:p.R79C is classified mostly as likely benign while TNNT2:p.R286H is reported mostly as a VUS. Computational evidence supports a potential pathogenic role as both variants are deleterious predictions by SIFT, Polyphen2 HumVar, MutationTaster, and both have scaled CADD scores of >30 (top 0.1%; Table VII in the Data Supplement).

**Table 3.**
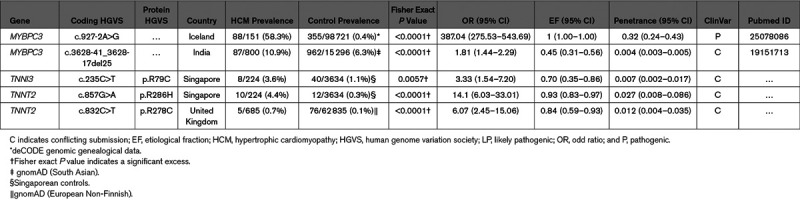
Comparison of Singaporean *TNNI3* and *TNNT2* Variants With Other Common HCM Variants of Reduced Penetrance

### Penetrance

Estimates of the population penetrance for TNNI3:p.R79C and TNNT2:p.R286H are both low (95% CI): 0.7% (0.2%–1.7%) and 2.7% (0.8%–8.6%), respectively (Table 3). These data are comparable with common HCM variants of low penetrance that have been reported in other populations.^11^ To assess segregation and study further the penetrance of TNNI3:p.R79C and TNNT2:p.R286H, we invited all families of the genotype-positive individuals from HCM cases and volunteer controls, and 10 families agreed to participate. The number of genotype-positive individuals identified in family studies was small, but analyzing both variants together the aggregate penetrance was 22.2% (2.8%–60.0%). Affected individuals in families studied were all >50, and many of the relatives assessed were younger (Figure III in the Data Supplement). There were insufficient affected relatives to robustly assess segregation, although there were no phenotype positive individuals who did not carry the variant. Interestingly, in one family the younger proband was compound heterozygous for TNNI3:p.R79C and TNNT2:p.R286H and had more severe left ventricular hypertrophy (20 mm) as compared to his elder sibling, who had the TNNI3:p.R79C variant only and relatively smaller LVMWT (15 mm) despite the older age (Figure IIIA in the Data Supplement).

Further family studies were conducted in Singaporean control subjects with TNNI3:p.R79C or TNNT2:p.R286H variants using cascade screening and cardiac magnetic resonance imaging. In Control Family 5, while the 30-years-old proband had no HCM or left ventricular hypertrophy, the 57-year-old father who also has the TNNI3:p.R79C variant was diagnosed with HCM with patchy fibrosis during screening as part of this study (Figure IIIB in the Data Supplement). The remaining 3 TNNI3:p.R79C families from the population controls and HCM cohort as well as the 5 TNNT2:p.R286H families from HCM and controls cohorts who agreed to participate in this study were uninformative for segregation analyses (Figure IIIC and IIID in the Data Supplement).

### Expressivity of Thin Filament Variants in the Population

**Figure 3.**
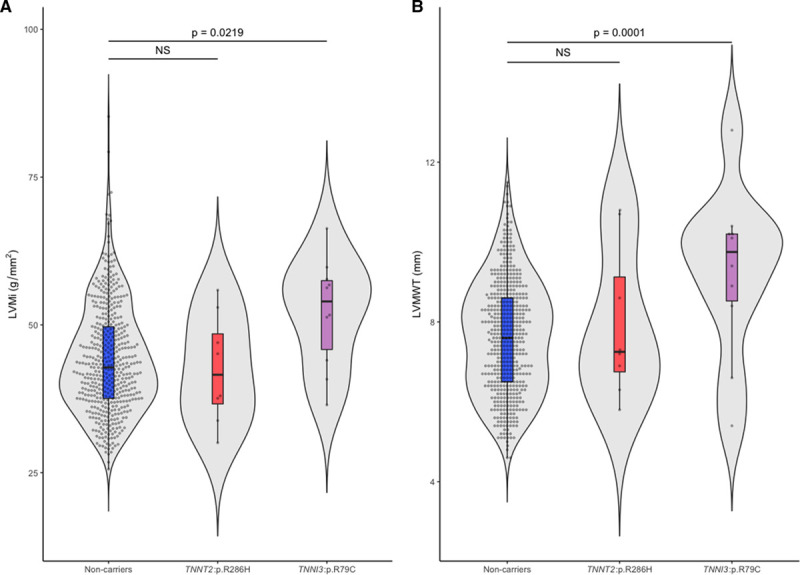
**TNNI3:p.R79C and TNNT2:p.R286H: Cardiac indices in the general population.** Violin plots comparing (**A**) indexed left ventricular (LV) mass (LVMi) and (**B**) left ventricular maximum wall thickness (LVMWT) in population controls with or without TNNI3:p.R79C or TNNT2:p.R286H, derived using cardiac magnetic resonance (CMR). Data were represented as median ± interquartile range (IQR) in a violin box-and-whiskers plot (Tukey rule) with the whiskers representing 1.5× IQR and outliers were plotted as individual dots. *P* values of regression models were derived using ANOVA where a significance cutoff of *P*<0.05 was used.

As measured by cardiac magnetic resonance, Singaporean population controls with TNNI3:p.R79C had significantly increased indexed LV mass (52.1 g/m^2^, *P*=0.0219) and LVMWT (9.2 mm, *P*=0.0001) when compared with the population controls without these variants (indexed LV mass, 44.1 g/m^2^; LVMWT=7.6 mm; Figure 3A and 3B; Table VIII in the Data Supplement). Carriers of TNNT2:p.R286H had similar LVMWT (8.0 mm, *P*>0.05) and indexed LV mass (42.6 g/m^2^, *P*>0.05) when compared with noncarrier individuals from the general population.

### Functional Analyses of iPSC-CMs With TNNT2:p.R286H

**Figure 4.**
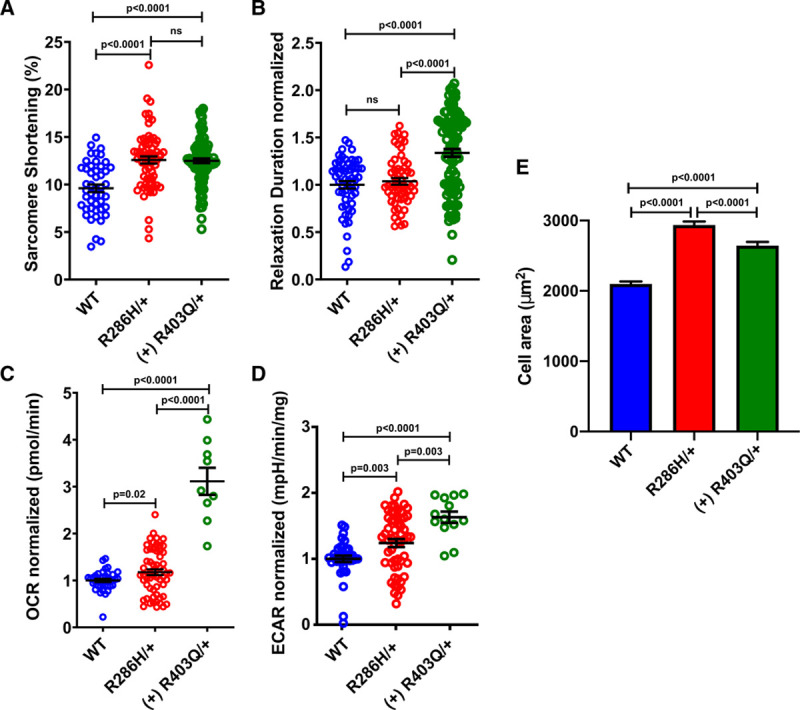
**Contractile characterization, metabolic flux, and cell size analysis of TNNT2:p.R286H iPSC-CMs (induced pluripotent stem cells derived cardiomyocytes).** **A**, Comparison of the percentage sarcomere shortening and (**B**) relaxation duration for isogenic wild type (WT), TNNT2:p.R286H (R286H/+) and established (+) hypertrophic variant of *MYH7*:p.R403Q (R403Q/+) iPSC-CMs. **C**, Measurement of oxygen consumption rate (OCR) and (**D**) extracellular acidification rate (ECAR) in WT, R286H/+ and R403Q/+ cardiomyocytes using the Seahorse platform and (**E**) unconstrained cell size in WT (n=586 cells), R286H/+ (n=408 cells) and (+) R403Q/+ (n=488 cells). All iPSC-CMs were generated by mutating an isogenic line, denoted TTN-GFP PGP1.^21,22^ Two or more differentiations were studied from 2 independent clones for each genotype. Data, mean ± SEM. Student *t* test for each mutant compared with WT was used where a significance cutoff of *P*<0.05 was used.

To examine the functional effect of TNNT2:p.R286H, we generated isogenic iPSC-CMs with and without the heterozygous TNNT2:p.R286H (R286H/+) variant as well as a pathogenic HCM variant—*MYH7*:p.R403Q (R403Q/+) for a positive control, as previously described.^25^ The contractile profiles, oxygen consumption rates, extracellular acidification rates, and cell size were examined. Assessment of sarcomere function (Figure 4A and 4B) showed that R286H/+ and R403Q/+ iPSC-CMs had similar significantly increased contractility (*P*<0.0001) in comparison to the wild type (WT). There was no significant change in the relaxation duration of R286H/+ iPSC-CMs while the R403Q/+ iPSC-CMs had significantly longer relaxation duration (*P*<0.0001) than R286H/+ iPSC-CMs and the WT. Significant increases in oxygen consumption rates (*P*=0.02) and extracellular acidification rate (*P*=0.003) were observed in R286H/+ iPSC-CMs compared with the WT. However, both parameters were significantly more abnormal (Figure 4C and 4D) in R403Q/+ iPSC-CMs when compared with the R286H/+ iPSC-CMs (oxygen consumption rates, *P*<0.0001; extracellular acidification rate, *P*=0.003) and WT (oxygen consumption rates and extracellular acidification rate, *P*<0.0001).

Additionally, the mean area of the unconstrained WT, R403Q/+ and R286H/+ were compared with determine the cellular hypertrophy in comparison to the WT (Figure 4E). Note that each of the mutant cells is significantly larger than WT cells (*P*<0.0001) while the R286H/+ are also larger than p.R403Q/+ (*P*<0.0001). We suggest that the hypercontractility, increased metabolic requirements, and cellular hypertrophy of the R286H/+ iPSC-CMs are consistent with cellular manifestations of HCM and, therefore, supports the likely pathogenicity of the TNNT2:p.R286H variant.

### Haplotype Analyses of TNNI3:p.R79C and TNNT2:p.R286H

To investigate the origins of these thin filament variants, we performed genome-wide genotyping of all carriers of TNNI3:p.R79C or TNNT2:p.R286H with or without HCM. We determined whether TNNI3:p.R79C and TNNT2:p.R286H are located on single haplotypes, suggesting a founder event, or multiple haplotypes suggestion more than one variant origin.^26–28^ We observed TNNI3:p.R79C to be in linkage disequilibrium with 2 common single-nucleotide polymorphisms (rs2288528 and rs2278281; Tables IX and X in the Data Supplement) suggesting that TNNI3:p.R79C is a founder variant in Chinese.

Since all carriers of TNNI3:p.R79C and TNNT2:p.R286H in our study were of Chinese origin except one potentially a mixed descendent of Chinese and Malay, we further explored the AF of both variants in different Chinese provinces (TNNI3:p.R79C only) and other East Asian countries using data extracted from the CONVERGE study (China, Oxford and VCU Experimental Research on Genetic Epidemiology) (n=11 670; Table XI in the Data Supplement), the Taiwan biobank (n=1517), the gnomAD-Korean (n=1908) and the human genetic variation database (Japanese, n=1207; Table 2). The AF of TNNI3:p.R79C in Fujian province located in Southeast China (0.0062), Taiwan (0.0060), South Korea (0.0063), and Japan (0.0041) were broadly similar to Singaporean (0.0055) yet relatively higher than other provinces in China (Figure IV in the Data Supplement). No common ancestral haplotype block was found for TNNT2:p.R286H suggesting it is a recurrent variant. The prevalence of TNNT2:p.R286H in Taiwanese population (AF=0.0013) is similar to Singaporean controls (AF=0.0017) while the AF for TNNT2:p.R286H in Chinese provinces was not available (Figure IV in the Data Supplement).

## Discussion

We used ACMG/AMP guidelines to identify disease variants for HCM in Singaporean patients. This revealed significantly fewer variants that could be robustly interpreted as P/LP and more VUSs in Singaporean HCM than reported in White HCM. These differences likely reflect the fact that White HCM has been very well studied, and there are deep White control data sets, whereas non-White HCM is relatively unstudied. We surmise that the fewer P/LP variants seen in Singaporean HCM is due to the fact that while disease-causing variants exist, they have not been identified as such. This is in keeping with the increased levels of excess of VUS seen in Singaporean HCM.

The discoveries of founder and recurrent pathogenic variants with high levels of penetrance are recognized and reported.^29–31^ However, incomplete penetrance occurs in HCM and current ACMG/AMP guidelines can inadequately classify variants. Low penetrance founder variants in *MYBPC3* are enriched in HCM cases in South Asian (c.3628-41_3628-17del25)^11^ and Icelandic (c.927-2A>G) populations.^12^ However, low penetrance HCM-related variants have not been reported previously in thin filament sarcomeric genes and TNNI3:p.R79C and TNNT2:p.R286H, which we describe here, are the first such reported.

Although there were a limited number of families for segregation studies, the observed aggregated penetrance in families (22.2%) was higher than the predicted combined population penetrance at 3.4% (0.7% [TNNI3:p.R79C]; 2.7% [TNNT2:p.R286H]). However, lifestyle, environmental factors,^32^ or modifier genes^33^ among family members can also contribute to apparent measures of variant penetrance in the families. Of note, during this study, we observed 5 White HCM cases with TNNT2:p.R278C from a UK HCM cohort (Table 3) with low penetranceth TNNT2:p.278C, 0.012 [95% CI, 0.004–0.035]) similar to the Chinese variants in *TNNT2* and *TNNI3* described here.

While the penetrance of the thin filament variants we describe here is low, they could account for a meaningful proportion of disease risk in patients with HCM who are found to carry these variants (etiological fraction of 0.93 [95% CI, 0.83–0.97] for TNNT2:R286H, etiological fraction of 0.70 [95% CI, 0.35–0.86] for TNNI3:p.R79C). Using current ACMG/AMP guideline and data reported here, the TNNT2:p.R286H variant could be reconsidered as LP instead of VUS when rule BS1 (the presence of the variant in Singaporean population controls) is revised with rule PS4 (significantly higher prevalence of the variant in Singaporean patients with HCM). The deleterious effects of TNNT2:p.R286H seen in the functional studies (rule PS3) further support a possible reclassification in Singapore. Meanwhile, TNNI3:p.R79C variant might be reclassified as VUS from Benign/Likely Benign (8 studies in ClinVar, Table VII in the Data Supplement) when rule BS1 and PP3 are activated.

Our functional studies of iPSC-CMs encoding TNNT2:p.R286H showed hypercontractility, cellular hypertrophy, and higher metabolic requirements than isogenic WT iPSC-CMs. These parameters are also abnormal in isogenic iPSC-CMs encoding the definitive HCM pathogenic variant *MYH7*:p.R403Q, supporting our conclusion that TNNT2:p.R286H is a likely pathogenic variant. However, we note that the TNNT2:p.R286H variant did not perturb relaxation times, unlike the MYH7:p.R403Q variant, and that all of the parameters studied were significantly more abnormal in *MYH7*:p.R403Q than in TNNT2:p.R286H. These differences may imply distinct mechanisms by which thick and thin filament pathogenic variants cause HCM. For example, abnormal sarcomere function might occur from thick filament variants by their influence on the conformational states of myosin,^25^ while thin filament variants may alter sarcomere function by influencing calcium sensitivity. Irrespective of this or other mechanisms that account for these differences, we suggest that normal relaxation and attenuated dysfunction of other parameters that we observed in TNNT2:R286H iPSC-CMs and could account for milder phenotype and reduced disease penetrance among HCM patients with this variant.

A limitation of our study is that we did not functionally validate the TNNI3:pR79C variant, which will be reported in follow-on studies. We noted elevated left ventricular mass and wall thickness in the general population with the TNNI3:pR79C variant, which requires further study. Given the size of the Chinese population, the *TNNT2* and *TNNI3* variants described here could be associated with a theoretical 200 000 cases of HCM in China. Further work is needed to determine the implications of our findings given the penetrance of both variants is low for overt HCM and replication and extension studies are required to assign robustness for clinical interpretation.

## Acknowledgments

We thank Professor Jonathan Flint for sharing the data of CONVERGE study, all contributors to the SingHealth Exome Consortium (SEC), the healthy volunteers, patients, and families for participating in this study. We also thank clinical research coordinators for recruiting patients and families.

## Sources of Funding

The research was supported in part by the National Medical Research Council (NMRC) Singapore STaR awards (NMRC/STaR/0011/2012 and NMRC/STaR/0029/2017 to Dr Cook), Goh Foundation, and Tanoto Foundation, the NMRC Centre Grant and Collaborative Centre Grant schemes (NMRC/CGAug16C006 to the NHCS), NHCS Centre Grant Seed Funding (NHCS-CGSF/2018/001 to C.J.P), NIHR Imperial College Biomedical Research Centre, Wellcome Trust (107469/Z/15/Z to Dr Ware), Wellcome Trust Sir Henry Wellcome fellowship (206466/Z/17/Z to Dr Toepfer), Medical Research Council (intramural awards to Drs Cook and Ware), Health Innovation Challenge Fund award from the Wellcome Trust and Department of Health (UK; HICF-R6-373; Drs Cook and Ware), the British Heart Foundation (SP/10/10/28431 to Dr Cook), BHF Centre of Research excellence Intermediate Transition Fellowship (Dr Toepfer), Sarnoff Foundation (Dr Garfinkel and G.G. Repetti), Fondation Leducq (Drs Cook, Seidman, and Seidman), São Paulo Research Foundation (FAPESP 2019/11821-1 to Dr Venturini). The views expressed in this work are those of the authors, and the funding institutions played no role in the design, collection, analysis, or interpretation of the data or in the decision to submit the manuscript for publication.

## Disclosures

None.

## Supplementary Material


