# Supplementary material for: Genetic Studies of Hypertrophic Cardiomyopathy in Singaporeans Identify Variants in TNNI3 and TNNT2 That Are Common in Chinese Patients
Source: Circ Genom Precis Med. 2020 Aug 20;13(5):424–34. doi: 10.1161/CIRCGEN.119.002823 (PMC7676617; doi:10.1161/CIRCGEN.119.002823)
Supplement: Supplementary file 1 [file hcg-13-424-s001.pdf]

SUPPLEMENTAL MATERIAL

ADDITIONAL METHODS

Subjects

Prospective unrelated patients with a diagnosis of HCM (n=224) as confirmed by cardiac magnetic resonance imaging (MRI) and/or echocardiographic diagnostic criteria were recruited from National Heart Centre Singapore (NHCS) (n=159) and National University Hospital Singapore (n=65). Singaporean controls (n=3,634) comprising of 1,000 unrelated individuals without cardiovascular diseases (CVD) or family history of CVD recruited at NHCS via advertisement and 2,634 aggregated genomes of self-reported healthy individuals from Singapore Exome Consortium (SEC)[1] were collected. Clinically annotated genetic data from 6,179 additional HCM cases, predominantly Caucasian, referred to the Oxford Molecular Genetics Laboratory (OMGL) or the Partners Laboratory for Molecular Medicine (LMM) were retrieved from Atlas of Cardiac Genetic Variation (ACGV) dataset[2-4]. All Singaporean participants gave written informed consent to participate in this ethics board approved study, which was carried out in accordance with local Tissue Acts.

Sequencing and variant classification of prospective hypertrophic cardiomyopathy patients

Genetic evaluation of variation in established HCM-associated genes was performed using targeted re-sequencing in all 224 index Singaporean HCM patients as previously described[5]. Variants of 15 genes[6] either robustly associated with HCM, or well-validated pheno/genocopies

(*ACTC1, CSRP3, FHL1, GLA, LAMP2, MYBPC3, MYH7, MYL2, MYL3, PLN, PRKAG2, TNNC1, TNNI3*, *TNNT2*, *TPM1*) were annotated using CardioClassifier, an inherited cardiac condition-specific decision-support tool[7]. Genome sequencing (GS) was performed in an additional 1,000 unrelated controls using the TruSeq DNA Nano kit (Illumina, California, US) according to manufacturer's instructions. The Illumina HiSeq X Ten system was then used to perform GS (2 X 150 bp) to a mean genome-wide read depth of 20X with at least 80% of bases covered >20X. The AF of *TNNI3*:p.R79C (ENST00000344887:c.235C>T) and *TNNT2*:p.R286H (ENST00000367318:c.857G>A) in Singaporean reference controls (n=3,634) were retrieved from the combined GS data and the aggregated genomes from SEC.

Population reference data

Genome Aggregation Database (gnomAD) is a reference population dataset comprising 125,748 exomes and 15,708 genomes from unrelated individuals from 8 major populations including East Asian and Caucasian[8]. Data were downloaded from gnomAD website (http://gnomad.broadinstitute.org, Oct 2018) [version 2.1, Oct 2018] containing information about the sub-continental populations in Europe and East Asia. In addition, allele frequencies of *TNNI3*:p.R79C and *TNNT2*:p.R286H were retrieved from Taiwan Biobank (https://taiwanview.twbiobank.org.tw/index, Oct 2018; n=1,517 Taiwanese controls[9]) and the Human Genetic Variation Database (HGVD, http://www.hgvd.genome.med.kyoto-u.ac.jp/index.html, July 2019; n=1,208 Japanese controls[10]). AF of *TNNI3*:p.R79C was also retrieved from the CONVERGE dataset representing 11,670 female Han Chinese controls across 24 provinces of China but not TNNT2:p.R286H which was not included in the CONVERGE study[11].

Genetic variation between HCM and controls

Protein-altering variants (annotated as predicted missense, nonsense, frameshift, inframe indels and essential splice site) in designated canonical transcripts with high quality score (PASS) were studied (Table S1). Globally rare variants, defined by a gnomAD global minor allele frequency (MAF) <0.0001 (based on the reasoning from Walsh et al[4]), were included in analysis, taking this threshold to define an inclusive variant set to take forward for further annotation and review. As the disease architecture of HCM in Singaporean predominantly Chinese is not well-characterised, low frequency variants as defined by a gnomAD global MAF <0.001 were assessed for possible enrichment in the local index HCM patients compared to gnomAD population controls using Fisher's Exact test and manually curated using ACMG/AMP guidelines in the study (Table S2). The case excess (likely contribution of variants in a gene to disease) was estimated by combining the proportion of pathogenic and likely pathogenic (P/LP) variants along with the excess of VUS (exVUS)in index cases compared with the population background as previously described[2]. Fisher's exact test with Bonferroni correction (n=15 genes, P<0.0033) was used to test an association between rare variant prevalence and disease status. Odds ratio (OR) and etiological fraction (EF) with 95% confidence intervals (CI) were calculated as previously described[12, 13].

Penetrance estimation

The population penetrance of each disease variant (*TNNI3*:p.R79C and *TNNT2*:p.R286H) was estimated as described previously (https://www.cardiodb.org/allelefrequencyapp/)[14], using prevalence of the disease equals 1 in 500, allelic frequency of the variants among HCM patients and population reference controls retrieved from ethnicity specific databases. Additionally, the penetrance in family members was estimated by calculating the proportion of genotype positive relatives found to manifest disease. Index cases were not included in the calculation, whether ascertained as cases or controls. Briefly, first degree relatives of the index Singaporean HCM patients and Singaporean controls with *TNNI3*:p.R79C or *TNNT2*:p.R286H variants were invited to participate in family studies on a research basis by the presence of *TNNI3*:p.R79C or *TNNT2*:p.R286H and a phenotype meeting HCM criteria. In the Singaporean HCM cohort where the probands are clinically diagnosed with HCM, 2 families with *TNNI3*:p.R79C and 3 families with *TNNT2*:p.R286H agreed to participate. Similarly, in the Singaporean control cohort where the index controls did not have HCM, 3 families with *TNNI3*:p.R79C and 2 families with *TNNT2*:p.R286H were recruited. All subjects were assessed using CMR imaging or echocardiography in combination with evaluation of HCM gene variation using TruSight Cardio panel similar to Singaporean HCM patients.

Genotyping and haplotype analysis

A subset of Singaporean HCM (n = 114) and controls (n = 596) were genotyped using Illumina Infinium OmniExpress-24 kit version 1.1 to assess for evidence of population stratification. Additionally, to investigate the origin of thin filament encoding variants (*TNNI3*:p.R79C and *TNNT2*:p.R286H), carriers with these variants (including Singaporean HCM patients, controls and family members who participated in family

studies) were genotyped using Illumina Infinium OmniExpress-24 kit version 1.1 or 1.2 according to the manufacturer’s protocols. Genotype clustering was performed using Illumina Genome Studio 2.0 software and low-quality samples with overall call rate of <0.99 were repeated. Principal-component analysis (PCA) was performed to measure the degree of genetic stratification and population substructure from different ethnicities as previously described[15-17]. Principal-component plots were generated using the GraphPad PRISM version 7.04 software (California, US).

Cardiac magnetic resonance (CMR) and echocardiography imaging.

Participants including all Singaporean HCM patients, family members and a subset of Singaporean Chinese controls (n=492/1,000, 49%) recruited from year 2014 to 2016 were investigated using CMR or echocardiography imaging. Participants underwent cine balanced steady-state free precession (b-SSFP) CMR imaging on at 1.5T (Aera, Siemens, Erlangen, Germany) or 3T (Ingenia, Philips, Best, Netherlands). Analysis of the CMR scans was carried out by experienced cardiologists, blinded to genotyping data, using commercially available semi-automated software (CMR42 software, Circle Cardiovascular Imaging, Alberta, Canada) and standardized protocols. The diagnostic criterion of HCM in Singaporean HCM patients and family members was defined as having a maximum left ventricular wall thickness of ≥13 mm (local clinical criteria) by CMR or echocardiography.

Engineering of TNNT2*:p.R286H *variant by CRISPR Cas9 into isogenic human iPSC

TNNT2:p.R286H was engineered into isogenic human iPSCs containing GFP-tag on the amino terminus of titin. GFP-TTN iPSCs were nucleofected using CRISPR/Cas9 and a 20-basepair guide (5'-CCGCGACCTTTATCTCGGAC-3') targeting the *TNNT2* gene as previously described[18]. Isolated subclones were purified and sequenced by Sanger sequencing and MiSeq (>500 reads) to confirm a heterozygous base substitution, G>A, in the 12th codon of *TNNT2* exon 16, which encodes *TNNT2*:p.R286H (Figure S5). An established heterozygous pathogenic HCM variant (*MYH7*:p.R403Q) was engineered similarly as positive control[19].

Assessment of sarcomere function in iPSC-CMs

Cardiomyocytes were differentiated from induced pluripotent stem cells (iPSC-CMs) for engineered lines and isogenic controls as described[20]. GFP-tagged iPSC-CMs were re-plated into 12-well plates containing 1:100 Matrigel (Corning) in RPMI 1640 medium at day 14 post-differentiation. Two days later cells were returned to RPMI containing B27 with insulin, and media changed every two days. Imaging was performed at day 30 post-differentiation of iPSC-CMs using SarcTrack as described[21].

Seahorse assay

iPSC-CMs ($12x10^4$ cells) were seeded onto a Matrigel-coated XF96 plate and studied using a Seahorse XF96 Analyzer (Agilent Technologies, Waldbronn, Germany). The Cell Mito Stress Kit was used to measure cellular oxygen consumption rate (OCR) and extracellular acidification

rate (ECAR) according to the manufacturer's instructions. Cells were washed with prewarmed XF media (non-buffered DMEM supplemented with 2 mM sodium pyruvate, 4mM L-glutamine and 10 mM glucose, pH 7.4) and incubated at 37 °C for 60 min without CO_2. Basal levels of OCR and ECAR were measured, followed by addition of 2 µM of oligomycin, 2 µM FCCP, 0.5 µM rotenone/0.5 µM antimycin A in a mitochondrial stress test. After the assay, cells were lysed with RIPA buffer and protein content was measured by BCA Assay (Pierce). Data were analyzed by WAVE software (Agilent).

iPSC-CM size assessment

Cell sizes were assessed after incubation with media containing 5µg/ml Wheat Germ Agglutinin, Alexa Fluor™ 568 (ThermoFisher Scientific) for 10 minutes at 37°C. After washing, samples were imaged using Nikon Ti Eclipse epifluorescence microscope and ≥10 images acquired from 10 regions for analyses using MATLAB program (The MathWorks, Natick, MA) that quantified pixel intensity units between fluorescent cells and the dark background. Data presented are from three separate differentiations, including > 400 cells per genotype. The mean and SEM were assessed and significance was measured by Student's t-test with a significance cut-off of $p<0.05$.

Statistical analysis

GraphPad PRISM version 7.04 software was used to perform Fisher's exact test with Bonferroni correction for multiple testing (n=15 genes, $p<0.0033$), pairwise correlation, parametric Student *t* test, one-way ANOVA or non-parametric Mann-Whitney U test and Kruskal-Walles test

depending on the normality of the data as assessed using Shapiro-Wilk test. Data is reported as means ± standard deviation or median (interquartile range) unless otherwise stated. Multivariate linear regression was performed using R version 3.6.0 (Boston, Massachusetts) to investigate the association between genotype and cardiac indices. Healthy Singaporean Chinese carriers of *TNNI3*:p.R79C (n=10) or *TNNT2*:p.R286H (n=8) genotypes and non-carriers (n=482) were included in the analysis. Models were adjusted for known clinical covariates (age, gender and systolic blood pressure) and significance was calculated using ANOVA. Statistical significance was taken with or without multiple comparison corrections and a significance cut-off of $p<0.05$.

Data Availability

All supporting data are available either within the article and the Data Supplement or will be available on a reasonable request to the corresponding author due to privacy issue and national laws under the provision that data may not leave the hospital/center premises.

REFERENCES:

1. Bylstra Y, Kuan JL, Lim WK, Bhalshankar JD, Teo JX, Davila S, Teh BT, Rozen S, Tan EC, Liew WKM, *et al.* Population genomics in South East Asia captures unexpectedly high carrier frequency for treatable inherited disorders. *Genet Med*. 2019;21:207-212.

2. Walsh R, Thomson KL, Ware JS, Funke BH, Woodley J, McGuire KJ, Mazzarotto F, Blair E, Seller A, Taylor JC, *et al.* Reassessment of Mendelian gene pathogenicity using 7,855 cardiomyopathy cases and 60,706 reference samples. *Genet Med*. 2017;19:192-203.

3. Alfares AA, Kelly MA, McDermott G, Funke BH, Lebo MS, Baxter SB, Shen J, McLaughlin HM, Clark EH, Babb LJ, *et al.* Results of clinical genetic testing of 2,912 probands with hypertrophic cardiomyopathy: expanded panels offer limited additional sensitivity. *Genet Med*. 2015;17:880-8.

4. Walsh R, Buchan R, Wilk A, John S, Felkin LE, Thomson KL, Chiaw TH, Loong CCW, Pua CJ, Raphael C, *et al.* Defining the genetic architecture of hypertrophic cardiomyopathy: re-evaluating the role of non-sarcomeric genes. *Eur Heart J*. 2017;38:3461-3468.

5. Pua CJ, Bhalshankar J, Miao K, Walsh R, John S, Lim SQ, Chow K, Buchan R, Soh BY, Lio PM, *et al.* Development of a Comprehensive Sequencing Assay for Inherited Cardiac Condition Genes. *J Cardiovasc Transl Res*. 2016;9:3-11.

6. Ingles J, Goldstein J, Thaxton C, Caleshu C, Corty EW, Crowley SB, Dougherty K, Harrison SM, McGlaughon J, Milko LV, *et al.* Evaluating the Clinical Validity of Hypertrophic Cardiomyopathy Genes. *Circ Genom Precis Med*. 2019;12:e002460.

7. Whiffin N, Walsh R, Govind R, Edwards M, Ahmad M, Zhang X, Tayal U, Buchan R, Midwinter W, Wilk AE, *et al.* CardioClassifier: disease- and gene-specific computational decision support for clinical genome interpretation. *Genet Med*. 2018.

8. Karczewski KJ, Francioli LC, Tiao G, Cummings BB, Alföldi J, Wang Q, Collins RL, Laricchia KM, Ganna A, Birnbaum DP, *et al.* Variation across 141,456 human exomes and genomes reveals the spectrum of loss-of-function intolerance across human protein-coding genes. *bioRxiv*. 2019:531210.

9. Chen CH, Yang JH, Chiang CWK, Hsiung CN, Wu PE, Chang LC, Chu HW, Chang J, Song IW, Yang SL, *et al.* Population structure of Han Chinese in the modern Taiwanese population based on 10,000 participants in the Taiwan Biobank project. *Hum Mol Genet*. 2016;25:5321-5331.

10. Higasa K, Miyake N, Yoshimura J, Okamura K, Niihori T, Saitsu H, Doi K, Shimizu M, Nakabayashi K, Aoki Y, *et al.* Human genetic variation database, a reference database of genetic variations in the Japanese population. *J Hum Genet*. 2016;61:547-53.

11. Cai N, Bigdeli TB, Kretzschmar WW, Li Y, Liang J, Hu J, Peterson RE, Bacanu S, Webb BT, Riley B, *et al.* 11,670 whole-genome sequences representative of the Han Chinese population from the CONVERGE project. *Sci Data*. 2017;4:170011.

12. Cole P and MacMahon B. Attributable risk percent in case-control studies. *Br J Prev Soc Med*. 1971;25:242-4.

13. Robins JM and Greenland S. Estimability and estimation of excess and etiologic fractions. *Stat Med*. 1989;8:845-59.

14. Minikel EV, Vallabh SM, Lek M, Estrada K, Samocha KE, Sathirapongsasuti JF, McLean CY, Tung JY, Yu LP, Gambetti P, *et al.* Quantifying prion disease penetrance using large population control cohorts. *Sci Transl Med*. 2016;8:322ra9.

15. Raychaudhuri S, Iartchouk O, Chin K, Tan PL, Tai AK, Ripke S, Gowrisankar S, Vemuri S, Montgomery K, Yu Y, *et al.* A rare penetrant mutation in CFH confers high risk of age-related macular degeneration. *Nat Genet*. 2011;43:1232-6.

16. Aung T, Ozaki M, Lee MC, Schlotzer-Schrehardt U, Thorleifsson G, Mizoguchi T, Igo RP, Jr., Haripriya A, Williams SE, Astakhov YS, *et al.* Genetic association study of exfoliation syndrome identifies a protective rare variant at LOXL1 and five new susceptibility loci. *Nat Genet*. 2017;49:993-1004.

17. Jin SC, Homsy J, Zaidi S, Lu Q, Morton S, DePalma SR, Zeng X, Qi H, Chang W, Sierant MC, *et al*. Contribution of rare inherited and de novo variants in 2,871 congenital heart disease probands. *Nat Genet*. 2017;49:1593-1601.

18. Sharma A, Toepfer CN, Ward T, Wasson L, Agarwal R, Conner DA, Hu JH and Seidman CE. CRISPR/Cas9-Mediated Fluorescent Tagging of Endogenous Proteins in Human Pluripotent Stem Cells. *Curr Protoc Hum Genet*. 2018;96:21.11.1-21.11.20.

19. Toepfer CN, Garfinkel AC, Venturini G, Wakimoto H, Repetti G, Alamo L, Sharma A, Agarwal R, Ewoldt JF, Cloonan P, *et al*. Myosin Sequestration Regulates Sarcomere Function, Cardiomyocyte Energetics, and Metabolism, Informing the Pathogenesis of Hypertrophic Cardiomyopathy. *Circulation*. 2020.

20. Sharma A, Toepfer CN, Schmid M, Garfinkel AC and Seidman CE. Differentiation and Contractile Analysis of GFP-Sarcomere Reporter hiPSC-Cardiomyocytes. *Curr Protoc Hum Genet*. 2018;96:21.12.1-21.12.12.

21. Toepfer CN, Sharma A, Cicconet M, Garfinkel AC, Mucke M, Neyazi M, Willcox JAL, Agarwal R, Schmid M, Rao J, *et al*. SarcTrack. *Circ Res*. 2019;124:1172-1183.

22. Ma N, Zhang JZ, Itzhaki I, Zhang SL, Chen H, Haddad F, Kitani T, Wilson KD, Tian L, Shrestha R, *et al*. Determining the Pathogenicity of a Genomic Variant of Uncertain Significance Using CRISPR/Cas9 and Human-Induced Pluripotent Stem Cells. *Circulation*. 2018;138(23):2666-2681.

Additional Tables

Table S1. Gene and canonical transcripts analysed in Singaporean and UK/US (ACGV) HCM patients.

Gene	Gene name	Ensembl transcipt ID	Singaporean HCM Cases	UK/US HCM Cases
ACTC1	actin, alpha, cardiac muscle 1	ENST00000290378	224	4185
CSRP3	cysteine and glycine-rich protein 3 (cardiac LIM protein)	ENST00000533783	224	2167
FHL1	four and a half LIM domains 1	ENST00000370690	224	1535
GLA	galactosidase, alpha	ENST00000218516	224	3700
LAMP2	lysosomal-associated membrane protein 2	ENST00000200639	224	3290
MYBPC3	myosin binding protein C, cardiac	ENST00000545968	224	6179
MYH7	myosin, heavy chain 7, cardiac muscle, beta	ENST00000355349	224	6112
MYL2	myosin, light chain 2, regulatory, cardiac, slow	ENST00000228841	224	4185
MYL3	myosin, light chain 3, alkali; ventricular, skeletal, slow	ENST00000395869	224	4185
PLN	phospholamban	ENST00000357525	224	2167
PRKAG2	protein kinase, AMP-activated, gamma 2 non-catalytic subunit	ENST00000287878	224	3973
TNNC1	troponin C type 1 (slow)	ENST00000232975	224	632
TNNI3	troponin I type 3 (cardiac)	ENST00000344887	224	6047
TNNT2	troponin T type 2 (cardiac)	ENST00000367318	224	6103
TPM1	tropomyosin 1 (alpha)	ENST00000403994	224	4447

Table S2. Low frequency protein-altering variants as defined by a gnomAD global MAF <0.001 and ≥0.0001 in Singaporean HCM patients.

Gene	Ensembl transcipt ID	Coding HGVS	Protein HGVS	Disease Allele count	Disease Allele Number	Control Allele Count (gnomAD-EA)	Control Allele Number (gnomAD-EA)	Fisher's Exact p-value*	Classification	Remarks
CSRP3	ENST00000533783	c.16G>A	p.Gly6Arg	2	448	19	19948	0.1218	Likely Benign	
MYBPC3	ENST00000545968	c.1000G>A	p.Glu334Lys	2	448	65	19470	0.6841	Likely Benign	
MYL3	ENST00000395869	c.170C>A	p.Ala57Asp	4	448	11	19952	**<0.0001**	Likely Benign	Well-established functional studies show no deleterious effect[22]
PRKAG2	ENST00000287878	c.331C>A	p.Gln111Lys	1	448	35	18378	0.8739	Likely Benign	
TNNI3	ENST00000344887	c.235C>T	p.Arg79Cys	8	448	120	19312	**0.0062**	VUS	
TPM1	ENST00000403994	c.845C>G	p.Thr282Ser	1	448	49[†]	30422[†]	0.7454	Likely Benign	

*Protein-altering variants were annotated as missense, nonsense, frameshift, inframe indels and essential splice site with high quality score (PASS). EA, East Asian; HGVS, Human Genome Variation Society; *Fisher's exact p-value in bold (<0.0083) indicates a significant excess, corrected for multiple testing (n=6); [†]gnomAD-South Asian (SA) based on the proband's ethnicity*
[22]Ma N, Zhang JZ, Itzhaki I, Zhang SL, Chen H, Haddad F, Kitani T, Wilson KD, Tian L, Shrestha R, et al. Determining the Pathogenicity of a Genomic Variant of Uncertain Significance Using CRISPR/Cas9 and Human-Induced Pluripotent Stem Cells. Circulation. 2018;138(23):2666-2681.

Table S3. Singaporean HCM cases with pathogenic, likely pathogenic and VUS variants* in fifteen core HCM disease genes.

Gene	Ensembl transcipt ID	Variant (HGVS)	Variant (Protein)	Classification	Allele count
ACTC1	ENST00000290378	c.217A>G	p.I73V	VUS	1
ACTC1	ENST00000290378	c.301G>A	p.E101K	Likely Pathogenic	1
CSRP3	ENST00000533783	c.271C>G	p.Q91E	VUS	1
FHL1	ENST00000370690	c.613delG	p.Asp205ThrTer53	Likely Pathogenic	1
FHL1	ENST00000370690	c.736C>T	p.H246Y	VUS	1
GLA	ENST00000218516	c.644A>G	p.N215S	Likely Pathogenic	1
GLA	ENST00000218516	c.899T>C	p.L300P	Likely Pathogenic	1
GLA	ENST00000218516	c.1175G>C	p.R392T	VUS	1
MYBPC3	ENST00000545968	c.104G>A	p.R35Q	VUS	1
MYBPC3	ENST00000545968	c.118G>T	p.V40L	VUS	1
MYBPC3	ENST00000545968	c.224_228dupACCAG	p.Gly77ThrfsTer21	Likely Pathogenic	1
MYBPC3	ENST00000545968	c.329delC	p.Pro110LeufsTer49	Likely Pathogenic	1
MYBPC3	ENST00000545968	c.659A>G	p.Y220C	VUS	2
MYBPC3	ENST00000545968	c.761T>C	p.L254P	VUS	1
MYBPC3	ENST00000545968	c.772G>A	p.E258K	Pathogenic	1
MYBPC3	ENST00000545968	c.1021_1028delGGCGTCAC	p.G341X	Likely Pathogenic	1
MYBPC3	ENST00000545968	c.1038_1042dupCGGCA	p.Met348ThrfsTer4	Pathogenic	1
MYBPC3	ENST00000545968	c.1156G>T	p.E386X	Pathogenic	1
MYBPC3	ENST00000545968	c.1279T>C	p.S427P	VUS	1
MYBPC3	ENST00000545968	c.1471dupG	p.Val491GlyfsTer40	Likely Pathogenic	1
MYBPC3	ENST00000545968	c.1639delG	p.Val547CysfsTer8	Likely Pathogenic	1
MYBPC3	ENST00000545968	c.2336A>G	p.K779R	VUS	1
MYBPC3	ENST00000545968	c.2441_2443delAGA	p.Lys814del	VUS	1
MYBPC3	ENST00000545968	c.2519T>A	p.V840E	VUS	1
MYBPC3	ENST00000545968	c.2543C>T	p.A848V	VUS	2
MYBPC3	ENST00000545968	c.2678delC	p.Pro893GlnfsTer31	Likely Pathogenic	1
MYBPC3	ENST00000545968	c.2743G>T	p.E915X	Likely Pathogenic	1

MYBPC3	ENST00000545968	c.2905+1G>A	-	Pathogenic	1
MYBPC3	ENST00000545968	c.2915G>A	p.R972Q	VUS	1
MYBPC3	ENST00000545968	c.3148G>A	p.E1050K	VUS	1
MYBPC3	ENST00000545968	c.3179delT	p.Leu1060ArgfsTer15	Likely Pathogenic	1
MYBPC3	ENST00000545968	c.3215T>G	p.L1072R	VUS	1
MYBPC3	ENST00000545968	c.3217dupC	p.Arg1073ProfsTer4	Likely Pathogenic	1
MYBPC3	ENST00000545968	c.3624delC	p.Lys1209SerfsTer28	Pathogenic	2
MYBPC3	ENST00000545968	c.3673G>A	p.A1225T	VUS	1
MYBPC3	ENST00000545968	c.3712_3713delCT	p.Leu1238GlyfsTer3	Pathogenic	1
MYBPC3	ENST00000545968	c.3719T>C	p.I1240T	VUS	1
MYBPC3	ENST00000545968	c.3764C>A	p.A1255D	VUS	1
MYH7	ENST00000355349	c.371C>T	p.T124I	VUS	1
MYH7	ENST00000355349	c.427C>T	p.R143W	Likely Pathogenic	3
MYH7	ENST00000355349	c.727C>T	p.R243C	VUS	1
MYH7	ENST00000355349	c.985C>T	p.L329F	VUS	1
MYH7	ENST00000355349	c.1324C>T	p.R442C	Likely Pathogenic	1
MYH7	ENST00000355349	c.1956G>C	p.R652S	VUS	2
MYH7	ENST00000355349	c.1987C>A	p.R663S	Pathogenic	1
MYH7	ENST00000355349	c.1988G>A	p.R663H	Pathogenic	1
MYH7	ENST00000355349	c.2389G>A	p.A797T	Pathogenic	1
MYH7	ENST00000355349	c.2539A>G	p.K847E	Likely Pathogenic	1
MYH7	ENST00000355349	c.3094G>T	p.D1032Y	VUS	1
MYH7	ENST00000355349	c.3133C>T	p.R1045C	Likely Pathogenic	1
MYH7	ENST00000355349	c.3134G>A	p.R1045H	VUS	2
MYH7	ENST00000355349	c.3149G>A	p.R1050Q	VUS	1
MYH7	ENST00000355349	c.3200T>C	p.M1067T	VUS	1
MYH7	ENST00000355349	c.3743T>A	p.M1248K	VUS	1
MYH7	ENST00000355349	c.4348G>A	p.D1450N	VUS	1
MYH7	ENST00000355349	c.5134C>T	p.R1712W	Likely Pathogenic	1
MYH7	ENST00000355349	c.5240A>G	p.E1747G	VUS	1

MYH7	ENST00000355349	c.5704G>C	p.E1902Q	VUS	2
MYL2	ENST00000228841	c.49G>A	p.V17M	VUS	1
MYL3	ENST00000395869	c.92G>A	p.R31H	VUS	1
TNNC1	ENST00000232975	c.65C>A	p.A22E	VUS	1
TNNC1	ENST00000232975	c.430A>G	p.N144D	VUS	3
TNNI3	ENST00000344887	c.235C>T	p.R79C	VUS	8
TNNI3	ENST00000344887	c.307C>T	p.R103C	VUS	1
TNNI3	ENST00000344887	c.370G>C	p.E124Q	VUS	2
TNNI3	ENST00000344887	c.484C>T	p.R162W	Pathogenic	2
TNNI3	ENST00000344887	c.485G>A	p.R162Q	Likely Pathogenic	2
TNNI3	ENST00000344887	c.532A>G	p.K178E	Likely Pathogenic	1
TNNI3	ENST00000344887	c.596G>A	p.S199N	Likely Pathogenic	2
TNNT2	ENST00000367318	c.280C>T	p.R94C	Likely Pathogenic	1
TNNT2	ENST00000367318	c.388C>T	p.R130C	Likely Pathogenic	2
TNNT2	ENST00000367318	c.857G>A	p.R286H	VUS	10
TPM1	ENST00000403994	c.82G>C	p.D28H	VUS	1
TPM1	ENST00000403994	c.105G>C	p.R35S	VUS	2
TPM1	ENST00000403994	c.379A>C	p.M127L	VUS	1
TPM1	ENST00000403994	c.635A>T	p.E212V	VUS	1

*HGVS, Human Genome Variation Society; *Only protein-altering variants (annotated as missense, nonsense, frameshift, inframe indels and essential splice site) with high quality score (PASS) were studied*

Table S4. Prevalence of pathogenic, likely pathogenic variants and exVUS in fifteen major HCM genes in Singaporean HCM patients.

Gene	Cases sequenced	SG HCM			gnomAD	Fisher's exact p-value[§]	SG HCM exVUS[†] (%)	SG HCM Total Case Excess[‡] (%)
		P/LP frequency (%)	VUS frequency (%)	Total (%)	Very Rare* control VUS frequency (%)			
**Sarcomeric genes**								
MYBPC3	224	7.1	7.6	14.7	2.6	**<0.0001**	5.0	12.1
MYH7	224	4.5	6.7	11.2	2.1	**<0.0001**	4.6	9.1
TNNI3	224	3.1	4.9	8.0	0.3	**<0.0001**	4.6	7.7
TNNT2	224	1.3	4.5	5.8	0.4	**<0.0001**	4.1	5.4
TPM1	224	0.0	2.2	2.2	0.1	**<0.0001**	2.1	2.1
TNNC1	224	0.0	1.8	1.8	0.1	**0.0001**	1.7	1.7
ACTC1	224	0.4	0.5	0.9	0.1	0.0239	0.4	0.8
MYL2	224	0.0	0.5	0.5	0.2	0.4067	0.3	0.3
MYL3	224	0.0	0.5	0.5	0.4	0.5447	0.1	0.1
**Other HCM genes**								
FHL1	224	0.4	0.5	0.9	0.2	0.0820	0.3	0.7
CSRP3	224	0.0	0.5	0.5	0.4	0.5645	0.1	0.1
PLN	224	0.0	0.0	0.0	0.0	1.0000	no excess	0.0
**Pheno/Genocopy genes**								
GLA	224	0.9	0.5	1.3	0.2	0.0106	0.3	1.2
LAMP2	224	0.0	0.0	0.0	0.3	1.0000	no excess	no excess
PRKAG2	224	0.0	0.0	0.0	0.7	0.4121	no excess	no excess
Total		17.7	30.7	48.3	8.0	**<0.0001**	23.7	41.4

*exVUS, excess of variant variants of unknown significance; P, pathogenic; LP, likely pathogenic; SG, Singaporean; *, MAF<0.0001; †, caseVUS-controlVUS; ‡, P/LP/exVUS variants combined; §, comparison between total SG HCM cases and very rare* VUS in gnomAD control; Fisher's exact p-value in bold (<0.0033) indicates a significant excess, corrected for multiple testing (n=15)*

Table S5. Baseline Clinical Characteristics of Singaporean HCM patients with or without pathogenic/likely pathogenic sarcomeric gene variants.

	Total	P/LP sarcomeric gene variants	Others	p-value
**Clinical Characteristics**				
No. of participants	224	37	187	-
Male, % (n)	80 (179)	76 (28)	81 (151)	n/s
Self-reported race, % (n)				
Chinese	78 (175)	92 (34)	75 (141)	-
Malay	6 (14)	3 (1)	9 (13)	-
Indian	11 (24)	3 (1)	13 (23)	-
Others	5 (11)	3 (1)	5 (10)	-
Mean age at recruitment, years (SD)	55.0 (13.4)	52.4 (14.2)	55.6 (13.3)	n/s
Mean office SBP, mmHg (SD)	129 (18)	122 (15)	130 (19)	**0.0045**
Mean office DBP, mmHg (SD)	72 (13)	71 (13)	73 (12)	n/s
**Cardiac magnetic resonance (CMR)**				
Individuals with CMR, % (n)	56 (125)	50 (19)	57 (106)	-
Mean LV max wall thickness, mm (SD)	19.2 (4.2)	21.2 (5.9)	18.8 (3.7)	n/s
Mean LV mass index, g/m^2 (SD)	92.3 (29.0)	85.6 (29.2)	93.6 (29.0)	n/s
**Echocardiography (Echo)**				
Individuals with Echo, % (n)	44 (99)	50 (18)	43 (81)	-
Mean LV max wall thickness, mm (SD)	20.0 (4.9)	20.5 (4.4)	19.9 (5.0)	n/s

P, pathogenic; LP, likely pathogenic; SD, standard deviation; n/s, not significant

Table S6. Clinical Characteristics of Singaporean HCM patients with TNNI3:p.R79C, TNNT2; p.R286H and other TNNI3 or TNNT2 with pathogenic/likely pathogenic variants

	Singaporean HCM Patients					
	**Total**	**TNNI3:p.R79C**	**TNNI3 (P/LP)**	**TNNT2:p.R286H**	**TNNT2 (P/LP)**	**ANOVA p-value**
**Clinical Characteristics**						
No. of participants	224	8	7	10	3	-
Male, % (n)	80 (179)	88 (7)	57 (4)	70 (7)	0	-
Self-reported race, % (n)						
Chinese	78 (175)	100 (8)	100 (7)	90 (9)	100 (3)	-
Malay	6 (14)	0	0	10 (1)	0	-
Indian	11 (24)	0	0	0	0	-
Others	5 (11)	0	0	0	0	-
Mean age at recruitment, years (SD)	55.0 (13.4)	59.1 (12.5)	44.0 (18.8)	56.5 (10.8)	57.7 (21.6)	n/s
Mean office SBP, mmHg (SD)	129 (18)	130 (15)	119 (8)	117 (24)	126 (25)	n/s
Mean office DBP, mmHg (SD)	72 (13)	76 (12)	68 (13)	64 (11)	71 (21)	n/s
**Cardiac magnetic resonance (CMR)**						
Individuals with CMR, % (n)	56 (125)	38 (3)	86 (6)	40 (4)	67 (2)	-
Mean LV max wall thickness, mm (SD)	19.2 (4.2)	18.0 (4.4)	22.3 (7.5)	18.3 (2.1)	21.5 (0.7)	n/s
Mean LV mass index, g/m^2 (SD)	92.3 (29.0)	130.1 (77.0)	79.9 (27.4)	90.2 (20.4)	94.0 (18.0)	n/s
**Echocardiography (Echo)**						
Individuals with Echo, % (n)	44 (99)	62 (5)	17 (1)	60 (6)	33 (1)	-
Mean LV max wall thickness, mm (SD)	20.0 (4.9)	19.6 (4.9)	25.0 (0)	19.0 (4.6)	15	n/s

P, pathogenic; LP, likely pathogenic; LV, left ventricular; SD, standard deviation; n/s, not significant

Table S7. Missense effect prediction by computational algorithms and ClinVar status of *TNNI3*; p.R79C (rs3729712) and *TNNT2*; p.R286H (rs141121678)

Variants	Missense Effect		ClinVar			
	**Computational Algorithms**	**Computational Prediction**	**No**	**Study Name**	**Clinical Significance**	**Last Evaluation Date**
***TNNI3*:p.R79C (rs3729712)**	SIFT	Deleterious	1	GeneDx	Likely benign	Jun 12, 2017
	Polyphen-VAR	Possibly damaging	2	Stanford Center for Inherited Cardiovascular Disease,Stanford University	Likely benign	Sep 6, 2017
	MutationTaster	Disease-causing	3	Laboratory for Molecular Medicine,Partners HealthCare Personalized Medicine	Benign	Nov 22, 2017
	CADD	34	4	Ambry Genetics	Likely benign	Dec 7, 2017
			5	Molecular Diagnostic Laboratory for Inherited Cardiovascular Disease,Montreal Heart Institute	Likely benign	-
			6	Center for Advanced Laboratory Medicine, UC San Diego Health,University of California San Diego	Likely benign	May 08, 2018
			7	Invitae	Likely benign	Oct 31, 2018
			8	Mendelics	Benign	May 28, 2019
***TNNT2*:p.R286H (rs141121678)**	SIFT	Deleterious	1	CSER _CC_NCGL, University of Washington	Likely pathogenic	Jun 1, 2014
	Polyphen-VAR	Probably damaging	2	Stanford Center for Inherited Cardiovascular Disease,Stanford University	Uncertain significance	Jan 28, 2016

MutationTaster	Disease-causing	3	Blueprint Genetics	Uncertain significance	Nov 22, 2017
CADD	32	4	Ambry Genetics	Uncertain significance	Jan 18, 2018
		5	Laboratory for Molecular Medicine,Partners HealthCare Personalized Medicine	Uncertain significance	Jul 16, 2018
		6	GeneDx	Uncertain significance	Oct 02, 2018
		7	Invitae	Likely pathogenic	Dec 18, 2018

SIFT: http://sift.jcvi.org; Polyphen: http://genetics.bwh.harvard.edu/pph2; MutationTaster: http://www.mutationtaster.org; CADD: http://cadd.gs.washington.edu/

Table S8. Baseline MRI Characteristics of Singaporean healthy Chinese carriers and non-carriers of *TNNI3*:p.R79C and *TNNT2:*p.R286H

	Singaporean Chinese				
	**Non-carriers**	***TNNI3*: p.R79C**	**p-value***	***TNNT2*: p.R286H**	**p-value***
**Clinical Characteristics**					
No. of participants	482	10	-	8	-
Male, % (n)	49 (236)	70 (7)	-	50 (4)	-
Recruitment methods % (n)					
Population Controls	100 (482)	80 (8)	-	33 (2)	-
Family members of probands	0	20 (2)	-	67 (6)	-
Mean age at recruitment, years (SD)	50.8 (15.5)	44.7 (13.4)	n/s	42.0 (15.5)	n/s
Mean office SBP, mmHg (SD)	134 (12)	144 (20)	n/s	125 (17)	n/s
Mean office DBP, mmHg (SD)	80 (20)	86 (12)	n/s	81 (15)	n/s
**Cardiac magnetic resonance**					
LVEF, %	62 (7)	60 (4)	n/s	63 (3)	n/s
iLVEDV, mL/m2 (SD)	71 (12)	76 (9)	n/s	66 (11)	n/s
iLVESV, mL/m2 (SD)	27 (8)	31 (5)	n/s	25 (3)	n/s
Maximum LV wall thickness, mm (SD)	7.6 (1.4)	9.2 (2.1)	**0.0001**	8.0 (1.9)	n/s
iLVM, g/m^2 (SD)	44.1 (9.0)	52.1 (9.2)	**0.0219**	42.6 (9.2)	n/s

*iLVEDV, incdexed left ventricular end diastolic volume; iLVESV, indexed left ventricular end systolic volume, iLVM, indexed left ventricular mass; LVEF, left ventricular; SD, standard deviation; n/s, not significant; *, comparisons of multivariate liner and regression models adjusted by gender, age and SBP between non-carriers and TNNI3:p.R79C or TNNT2:p.R286H using ANOVA ; p-value in bold indicates a significance*

Table S9. Haplotype analysis of *TNNI3*:p.R79C (rs3729712) in unrelated probands from Singaporean HCM and population controls

Haplotype	Patient ID	Cohort	rs2288528	*TNNI3*:p.R79C (rs3729712)	rs2278281
1	0002	HCM	T	+	G
1	0004	HCM	T	+	G
1	0022	HCM	T	+	G
1	0025	HCM	T	+	G
1	0043	HCM	T	+	G
1	0000479	HCM	T	+	G
1	0000526	HCM	T	+	G
1	0000638	HCM	T	+	G
1	0001	Control	T	+	G
1	0009	Control	T	+	G
1	0013	Control	T	+	G
1	0048	Control	T	+	G
1	0055	Control	T	+	G
1	0057	Control	T	+	G
1	0048	Control	T	+	G
1	00481	Control	T	+	G

Table S10. Pairwise correlation of genetic markers around *TNNI3*:p.R79C forming a T-A-G haplotype.

Haplotype	Actual Frequency	Expected Frequency under Linkage Equilibrium
rs2288528 (chr19:55667500) | TNNI3:p.R79C (chr19:55667616) (R^2=0.659)		
TA	0.267857	0.095663
AA	0.000000	0.172194
TG	0.089286	0.261480
AG	0.642857	0.470663
TNNI3:p.R79C (chr19:55667616) | rs2278281 (chr19:55668197) (R^2=0.671)		
AG	0.275862	0.099881
GG	0.086207	0.262188
AT	0.000000	0.175981
GT	0.637931	0.46195
rs2288528 (chr19:55667500) | rs2278281 (chr19:55668197) (R^2=1.000)		
TG	0.357143	0.127551
AG	0.000000	0.229592
TT	0.000000	0.229592
AT	0.642857	0.413265

Table S11. Demographics and minor allele frequency of *TNNI3*, p.R79C (rs3729712) in CONVERGE Study

Chromosome	Position	Provinces of China	A1	A2	MAC	Allele number	MAF
19	55667616	Anhui	A	G	0	102	0.0000
19	55667616	Beijing	A	G	1	826	0.0012
19	55667616	Chongqing	A	G	1	602	0.0017
19	55667616	Fujian	A	G	1	162	0.0062
19	55667616	Gansu	A	G	1	248	0.0040
19	55667616	Guangdong	A	G	1	1552	0.0006
19	55667616	Guangxi	A	G	0	34	0.0000
19	55667616	Hainan	A	G	0	8	0.0000
19	55667616	Hebei	A	G	0	830	0.0000
19	55667616	Heilongjiang	A	G	1	778	0.0013
19	55667616	Henan	A	G	0	1182	0.0000
19	55667616	Hubei	A	G	1	398	0.0025
19	55667616	Hunan	A	G	0	146	0.0000
19	55667616	Jiangsu	A	G	4	1730	0.0023
19	55667616	Jiangxi	A	G	2	610	0.0033
19	55667616	Jilin	A	G	0	436	0.0000
19	55667616	Liaoning	A	G	2	1768	0.0011
19	55667616	Shaanxi	A	G	2	1648	0.0012
19	55667616	Shandong	A	G	2	1036	0.0019
19	55667616	Shanghai	A	G	6	2992	0.0020
19	55667616	Shanxi	A	G	2	726	0.0028
19	55667616	Sichuan	A	G	0	650	0.0000
19	55667616	Tianjin	A	G	0	402	0.0000
19	55667616	Zhejiang	A	G	3	2362	0.0013

MAC, minor allele count; MAF, minor allele frequency

Additional Figures

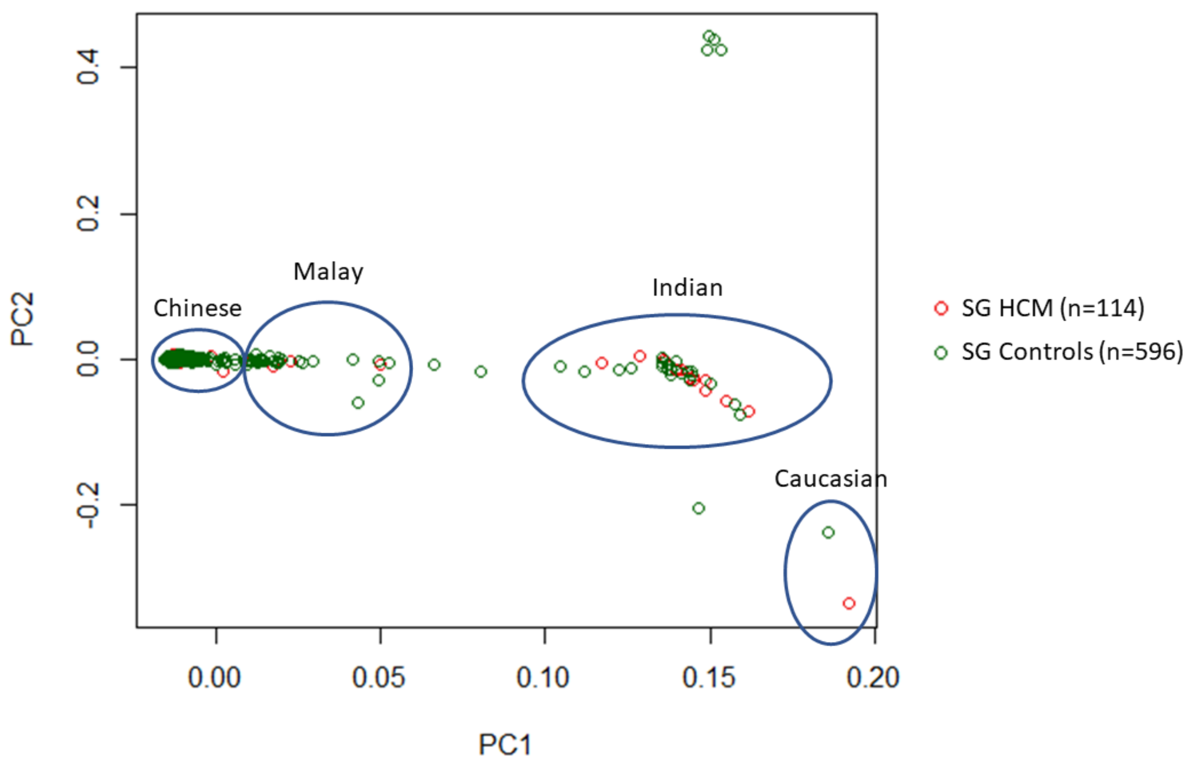

Figure S1. Population stratification of Singaporean HCM (red, n = 114) and controls (green, n = 596) using principal component analysis (PCA) showing overlapping clustering between both cohorts.

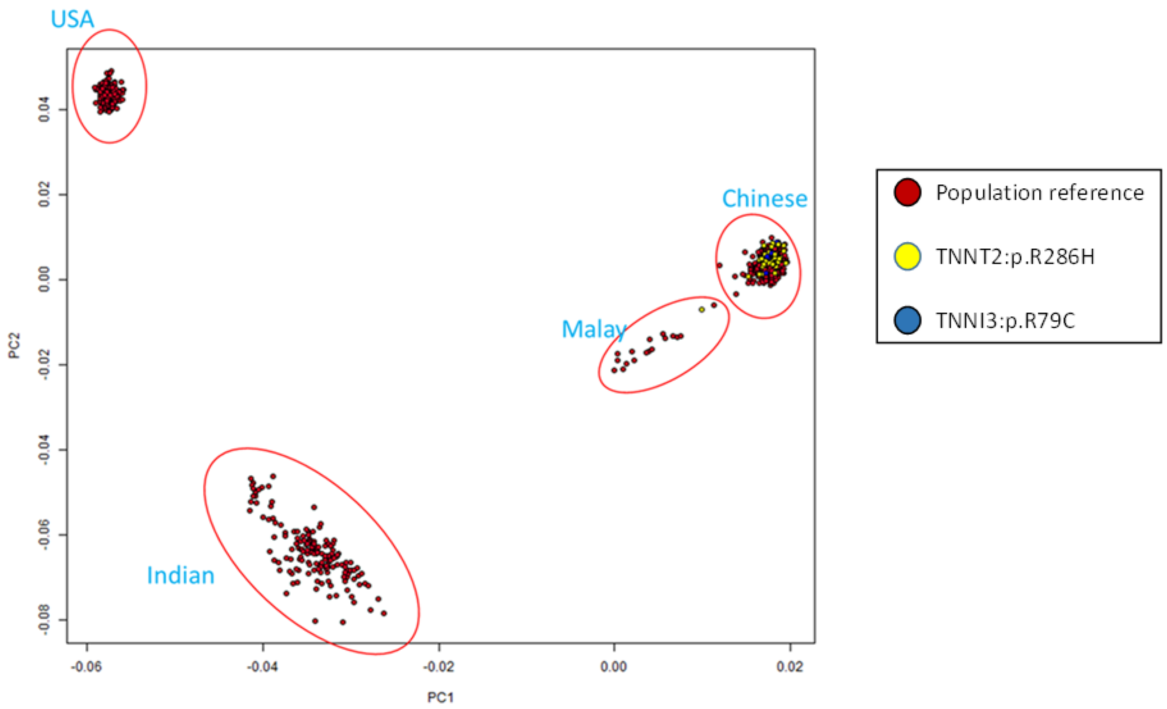

Figure S2. Principal component analysis for Singapore carriers of TNNI3:p.R79C (blue) or TNNT2:p.R286H variants (yellow) and control populations (red) representing Chinese, Malay, Indian and USA. All carriers except one were clustered under Chinese controls.

(a) **HCM Family 4**

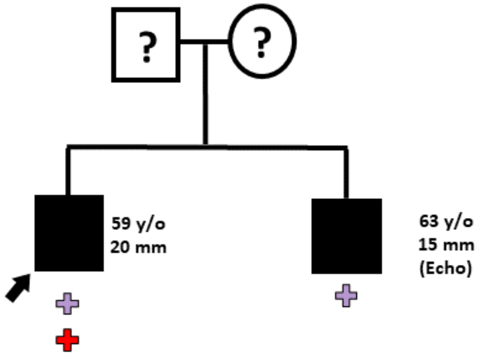

(b) **Control Family 5**

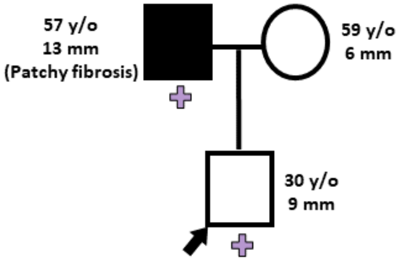

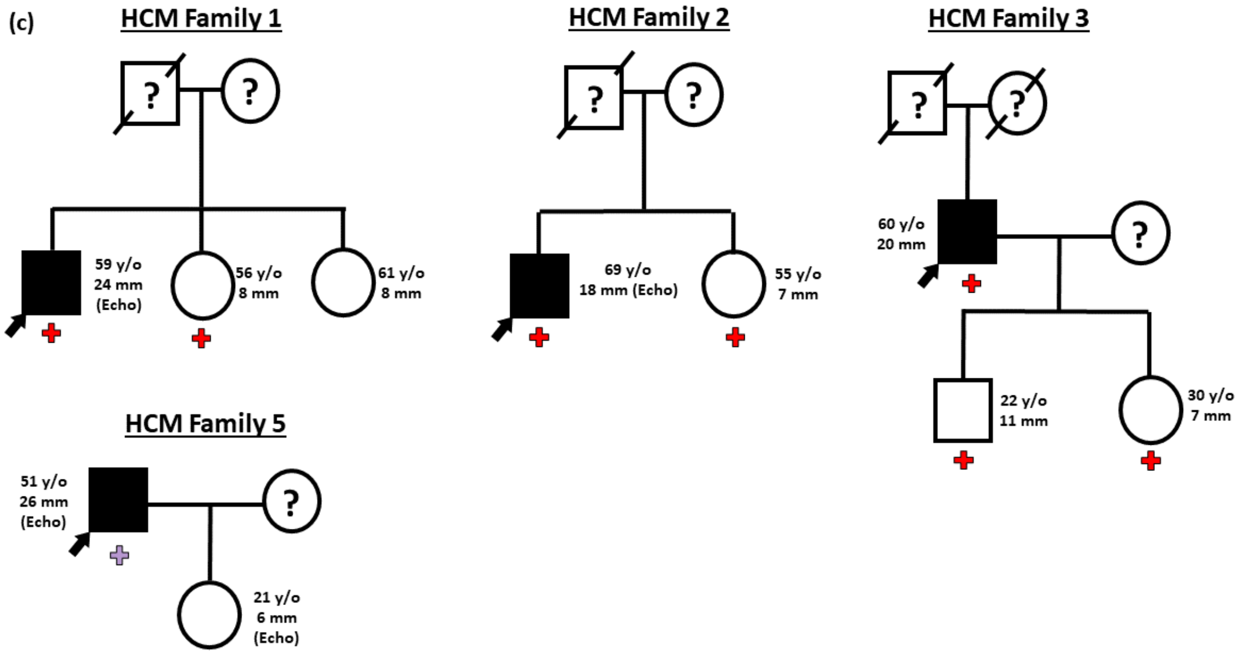
(c)
HCM Family 1
?
?
59 y/o
24 mm
(Echo)
56 y/o
8 mm
61 y/o
8 mm
HCM Family 2
?
?
69 y/o
18 mm (Echo)
55 y/o
7 mm
HCM Family 3
?
?
60 y/o
20 mm
?
22 y/o
11 mm
30 y/o
7 mm
HCM Family 5
51 y/o
26 mm
(Echo)
?
21 y/o
6 mm
(Echo)

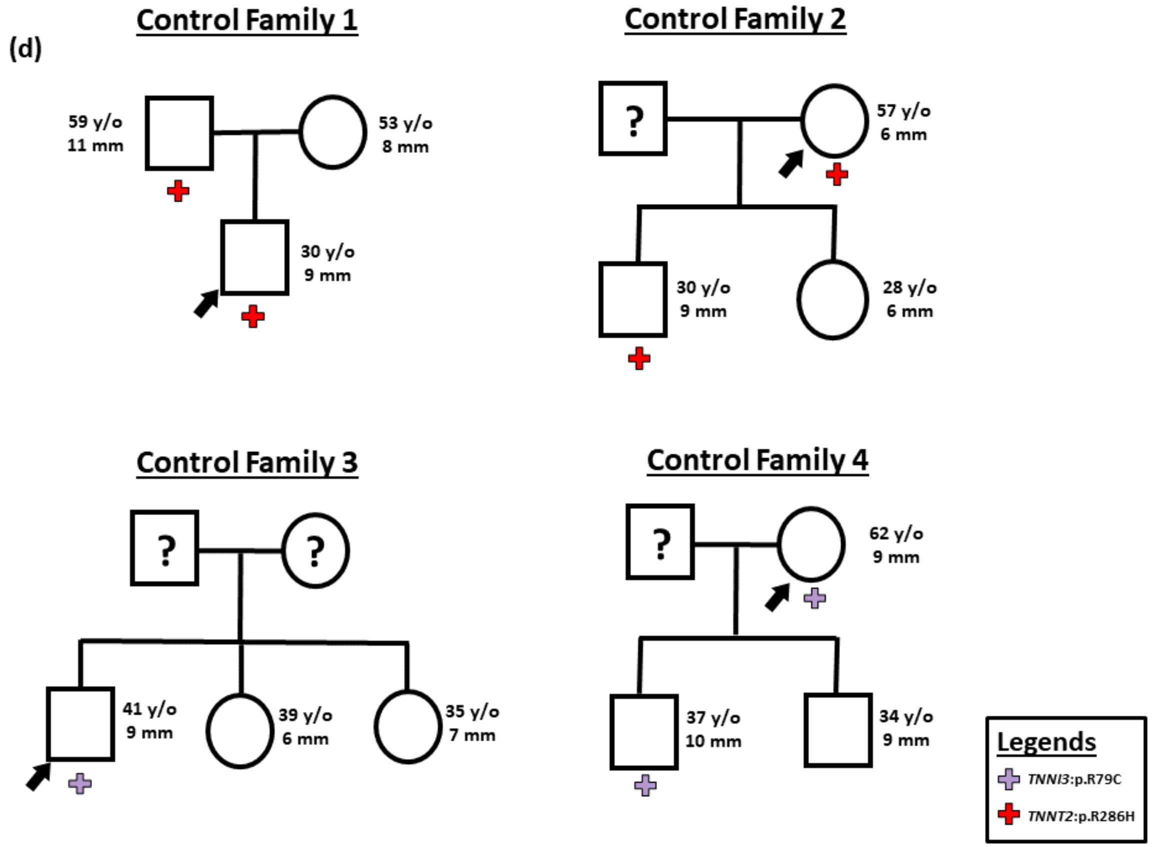

Figure S3. Family study of *TNNI3*:p.R79C and *TNNT2*:p.R286H recruited from Singaporean HCM cohort and local control cohort without family history of HCM. **a)** Segregation was observed in the HCM family 4 where the proband was compound heterozygous: TNNI3:p.R79C; TNNT2:p.R286H and the sibling was heterozygous for TNNI3:p.R79C. **b)** Population control Family 5, where the proband was recruited without family history of HCM. The father of proband who also has the TNNI3:p.R79C variant was diagnosed with HCM during this study. No left ventricular hypertrophy was observed in the carriers of *TNNI3*:p.R79C or *TNNT2*:p.R286H in the remaining **c)** HCM and **d)** control families.

Arrows: proband, **circles:** female, **squares:** male, **darkened**: LVH, **clear:** clinically unaffected, **slashed:** deceased, **interrogation (?):** genetic information was not available, **red plus (+):** *TNNT2*:p.R286H heterogeneous carriers, **purple plus (+):** *TNNI3*:p.R79C heterogeneous carriers.

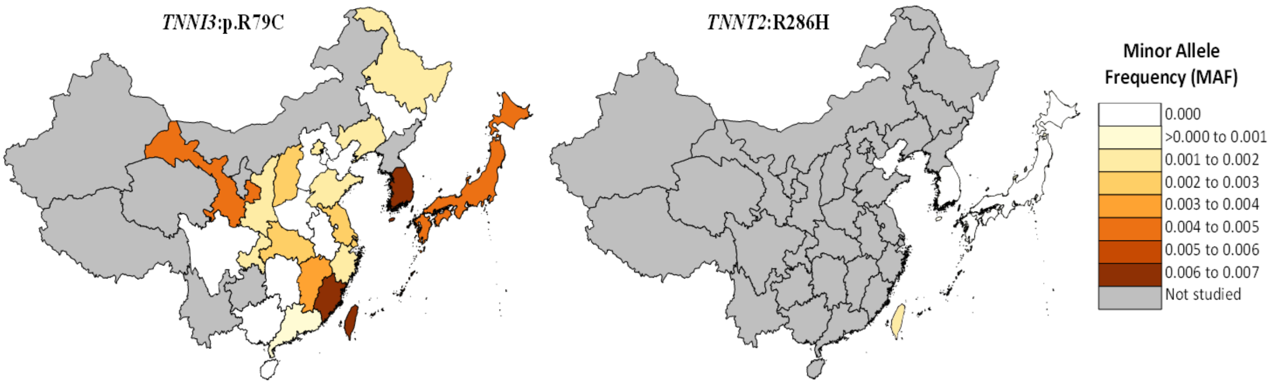

Figure S4. Allele Frequency (AF) of TNNI3:p.R79C and TNNT2:R286H in different provinces of China (CONVERGE Study), Taiwan (Taiwan Biobank), South Korea (gnomAD-EA-Korean) and Japan (Human Genetic Variation Database).

TNNT2 R286H - Heterozygous

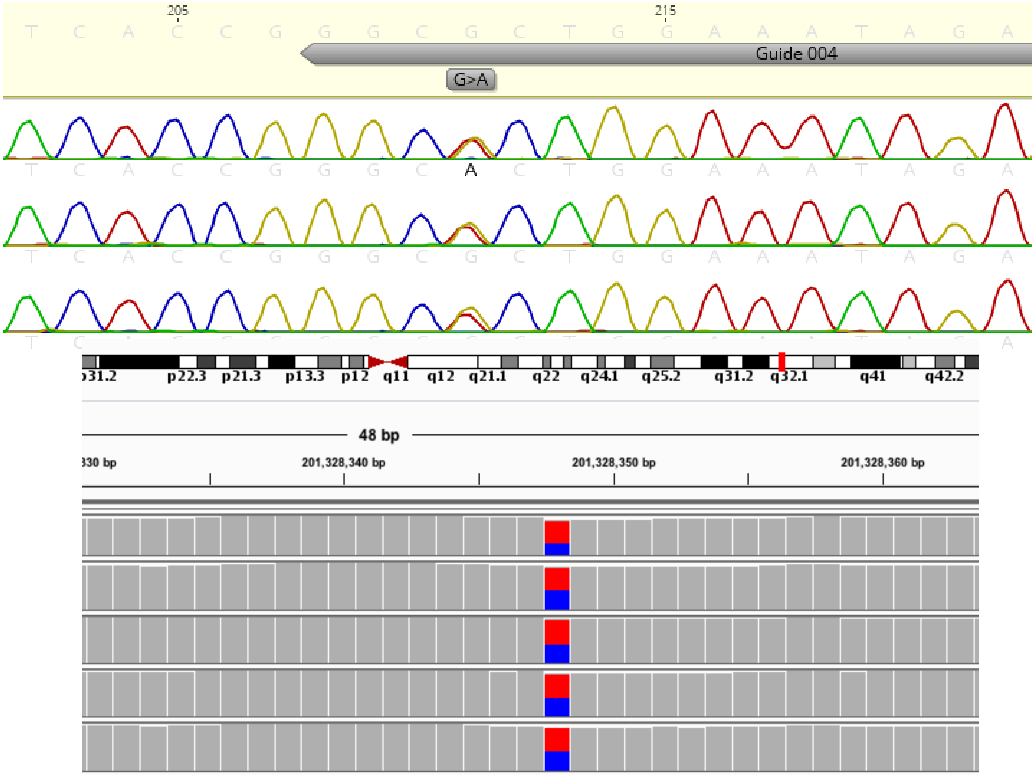

Figure S5. Sanger electropherograms of the *TNNT2:*p.R286H clones and heterozygous reads (n = >500 reads) by MiSeq. Both validation methods demonstrate that targeted mutagenesis resulted in a single missense residue from G>A in the 12th codon of TNNT2 exon 16, which encodes *TNNT2*:p.R286H.
