# Supplementary material for: Genetic Studies of Hypertrophic Cardiomyopathy in Singaporeans Identify Variants in TNNI3 and TNNT2 That Are Common in Chinese Patients
Source: Circ Genom Precis Med. 2020 Aug 20;13(5):424–34. doi: 10.1161/CIRCGEN.119.002823 (PMC7676617; doi:10.1161/CIRCGEN.119.002823)
Supplement: Supplementary file 2 [file hcg-13-424-s002.pdf]

Genetic Studies of Hypertrophic Cardiomyopathy in Singaporeans Identify Variants in *TNNI3* and *TNNT2* that Are Common in Chinese Patients

Running title: *Pua et al, TNNI3 and TNNT2 variants are common in Chinese HCM*

Chee Jian Pua, BSc[1,2]; Nevin Tham, BSc[1]; Calvin WL Chin, MD, PhD[1,3]; Roddy Walsh, PhD[4]; Chiea Chuen Khor, MBBS, PhD[5]; Christopher N. Toepfer, PhD[6,7]; Giuliana G. Repetti, BSc[6]; Amanda C. Garfinkel, MD[6]; Jourdan F. Ewoldt, BS[8]; Paige Cloonan, BS[8]; Christopher S. Chen, MD, PhD[8]; Shi Qi Lim, MSc[1]; Jiashen Cai, MD[3]; Li Yang Loo, MBBS[2]; Siew Ching Kong, BSc[1]; Charleston W. K. Chiang, PhD[9,10]; Nicola Whiffin, PhD [11,12]; Antonio de Marvao, PhD, MRCP[11,12]; Pei Min Lio, BSc[1]; An An Hii, BSc[1]; Cheng Xi Yang, BSc[1]; Thu Thao Le, PhD[1]; Yasmin Bylstra, FHGSA(GenCouns)[13]; Weng Khong Lim, PhD [13]; Jing Xian Teo, BSc[13]; Kallyandra Padilha, PhD[6,14]; Gabriela Venturini, PhD[6,14]; Bangfen Pan, MSc[15]; Risha Govind, MSc[11,12]; Rachel J Buchan, MSc[11,12]; Paul JR Barton, PhD[11,12]; Patrick Tan, MD, PhD[5,13]; Roger Foo, MD[5,15], James W.L. Yip, MRCP[16]; Raymond CC Wong, MRCP[16]; Wan Xian Chan, MRCP[16]; Alexandre C. Pereira, MD, PhD[6,14]; Hak Chiaw Tang, MRCP[1]; Saumya Shekhar Jamuar, MRCP[3,13,17,18]; ; James S. Ware MRCP, PhD[11,12]; Jonathan G. Seidman, PhD[6]*; Christine E. Seidman, MD[6,19]*; Stuart A. Cook, MRCP, PhD[1,3,11,12]*

[1]National Heart Ctr Singapore; [2]Yong Loo Lin School of Medicine, National Univ Singapore; [3]Duke-National Univ of Singapore Medical School, Singapore; [4]Dept of Clinical & Experimental Cardiology, Heart Ctr, Amsterdam Cardiovascular Sciences, Amsterdam UMC, Univ of Amsterdam, Amsterdam, the Netherlands; [5]Genome Inst of Singapore, Singapore; [6]Dept of Genetics, Harvard Medical School, Boston, MA; [7]Radcliffe Dept of Medicine, Univ of Oxford, Oxford, UK; [8]Dept of Biomedical Engineering, Boston Univ, Boston, MA; [9]Ctr for Genetic Epidemiology, Univ of Southern California; [10]Ctr for Neurobehavioral Genetics, Univ of California, Los Angeles, CA; [11]Cardiovascular Rsrch Ctr, Royal Brompton Hospital; [12]National Heart & Lung Institute, Imperial College London, London, UK; [13]SingHealth/Duke-NUS Precision Medicine Inst, Singapore; [14]Laboratory of Genetics & Molecular Cardiology, Heart Inst (InCor)-Univ of São Paulo Medical School, São Paulo, Brazil; [15]Cardiovascular Rsrch Inst, National Univ Health System; [16]Cardiology Dept, National Univ Heart Ctr Singapore; [17]KK Women's & Children's Hospital; [18]SingHealth Duke-NUS Genomic Medicine Ctr, Singapore; [19]Cardiovascular Division, Brigham & Women's Hospital Howard Hughes Medical Inst, Boston, MA

and Precision Medicine

Correspondence:

Stuart A. Cook, MRCP, PhD
Cardiovascular & Metabolic Disease Program
Duke-NUS Graduate Medical School
8 College Road, Level 8, Singapore 169857
E-mail: stuart.cook@singhealth.com.sg

Christine E. Seidman, MD
Department of Genetics
NRB Room 256, Harvard Medical School
77 Ave Louis Pasteur, Boston, MA 02115
E-mail: cseidman@genetics.med.harvard.edu

Jonathan G. Seidman, PhD
Department of Genetics
NRB Room 256, Harvard Medical School
77 Ave Louis Pasteur, Boston, MA 02115
E-mail: seidman@genetics.med.harvard.edu

Chee Jian Pua, BSc
National Heart Research Institute Singapore
55 National Heart Centre Singapore
5 Hospital Dr., Singapore 169609
E-mail: pua.chee.jian@nhcs.com.sg

Journal Subject Terms: Cardiomyopathy; Genetics; Heart Failure

Abstract:

Background - To assess the genetic architecture of hypertrophic cardiomyopathy (HCM) in patients of predominantly Chinese ancestry.

Methods - We sequenced HCM disease genes in Singaporean patients (n=224) and Singaporean controls (n=3,634), compared findings with additional populations and Caucasian HCM cohorts (n=6,179) and performed in vitro functional studies.

Results - Singaporean HCM patients had significantly fewer confidently interpreted HCM disease variants (Pathogenic (P)/Likely Pathogenic (LP):18%, $p<0.0001$) but an excess of variants of unknown significance (exVUS: 24%, $p<0.0001$), as compared to Caucasians (P/LP: 31%, exVUS: 7%). Two missense variants in thin filament encoding genes were commonly seen in Singaporean HCM (*TNNI3*:p.R79C, disease allele frequency (AF)=0.018; *TNNT2*:p.R286H, disease AF=0.022) and are enriched in Singaporean HCM when compared with Asian controls (*TNNI3*:p.R79C, Singaporean controls AF=0.0055, $p=0.0057$, gnomAD-East Asian (gnomAD-EA) AF=0.0062, $p=0.0086$; *TNNT2*:p.R286H, Singaporean controls AF=0.0017, $p<0.0001$, gnomAD-EA AF=0.0009, $p<0.0001$). Both these variants have conflicting annotations in ClinVar and are of low penetrance (*TNNI3*:p.R79C, 0.7%; *TNNT2*:p.R286H, 2.7%) but are predicted to be deleterious by computational tools. In population controls, *TNNI3*:p.R79C carriers had significantly thicker left ventricular walls compared to non-carriers while its etiological fraction is limited (0.70, 95% CI: 0.35-0.86) and thus *TNNI3*:p.R79C is considered a VUS. Mutant *TNNT2*:p.R286H iPSC-CMs show hypercontractility, increased metabolic requirements and cellular hypertrophy and the etiological fraction (0.93, 95% CI: 0.83-0.97) support the likely pathogenicity of *TNNT2*:p.R286H.

Conclusions - As compared to Caucasians, Chinese HCM patients commonly have low penetrance risk alleles in *TNNT2* or *TNNI3* but exhibit few clinically actionable HCM variants overall. This highlights the need for greater study of HCM genetics in non-Caucasian populations.

Circulation: Genomic and Precision Medicine

Key words: hypertrophic cardiomyopathy; troponin T; troponin I; Founder

Nonstandard Abbreviations and Acronyms

ACGV - Atlas of Cardiac Genetic Variation

ACMG/AMP - American College of Medical Genetics and Genomics and the Association for Molecular Pathology

AF - allele frequency

CI - confidence interval

CMR – cardiac magnetic resonance

EA – East Asian

ECAR - extracellular acidification rates

Echo - echocardiogram

EF - etiological fraction

exVUS – excess of variant of uncertain significance

gnomAD - Genome Aggregation Database

HCM – hypertrophic cardiomyopathy

HGVD – Human Genetic Variation Database

HGVS - Human Genome Variation Society

IQR - interquartile range

iPSC-CM - induced pluripotent stem cells derived cardiomyocytes

LP – likely pathogenic

LVH – left ventricular hypertrophy

LVMi - indexed left ventricular mass

LVMWT - left ventricular maximum wall thickness

OCR - oxygen consumption rates

OR – odds ratio

P – pathogenic

PCA - principal component analysis

SEC - SingHealth Exome Consortium

SG - Singaporean

SNP - single nucleotide polymorphisms

VUS – variant of uncertain significance

WT – wildtype

Circulation: Genomic and Precision Medicine

Introduction

Hypertrophic cardiomyopathy (HCM) is a common Mendelian disease with prevalence of up to 1 in 500 people[1] and is diagnosed by the presence of left ventricular hypertrophy (LVH) that cannot be explained by systemic or other cardiac diseases[2]. HCM is generally thought of as an autosomal dominant Mendelian disease of variable penetrance and expressivity where disease-causing variants, mostly with allele frequencies (AF) <0.0001, are found in up to 50% of patients[3]. *MYH7* was the first HCM gene identified[4] and rare variants are observed in approximately 13% of all HCM cases in one of the largest published cohorts (n=6,179) of HCM patients[3]. *MYBPC3* is the most prevalent HCM-associated gene and seen in approximately 17% cases, while other sarcomere-encoding genes (*ACTC1, MYL2, MYL3, TNNI3, TNNT2* and *TPM1*) account for most of the remaining cases with a genetic causation. Although more than 30 other genes are often included in HCM genetic tests[5], recent evaluation of these genes showed limited or no evidence of disease association for most of these genes[6].

Circulation: Genomic and Precision Medicine

The variant classification framework of the American College of Medical Genetics and Genomics and the Association for Molecular Pathology (ACMG/AMP) provides an evidence-based approach to classify the pathogenicity of sequence variants[7]. The release of publicly available Genome Aggregation Database (gnomAD) provides a reference population to refine our understanding of the spectrum of rare variation in the human genome, and to rule out alleles that are insufficiently rare to be causative of penetrant Mendelian disease[8, 9]. However, this approach needs to be used with caution as variants that are not very rare, including founder and recurrent variants, have been reported in HCM[10]. Previous studies identified two founder variants of *MYBPC3* that are significantly enriched in HCM cases in South Asian[11] and Icelandic populations[12], which are now mostly classified as pathogenic by clinical genetics laboratories in

ClinVar despite being relatively common in specific populations. It is important to have a firm understanding of the genetic architecture of HCM in a given population, and to recognize population specific alleles, including founder variants, before the ACMG/AMP framework can be confidently applied in that population[7].

In this study, we assessed the genetic architecture of HCM in Singapore in unrelated patients of predominantly Chinese ancestry. Variant calling and comparison with other HCM and control cohorts and the subsequent analyses of variants enriched in Singaporean HCM cases are outlined in a study overview cartoon in Figure 1.

Methods

American Heart Association.
Circulation: Genomic and Precision Medicine

We prospectively recruited unrelated Singaporean patients with HCM (n=224) and Singaporean controls (n=3,634)[13] and performed targeted re-sequencing[14]. Variants of 15 genes[6] either robustly associated with HCM, or well-validated pheno/genocopies (*ACTC1, CSRP3, FHL1, GLA, LAMP2, MYBPC3, MYH7, MYL2, MYL3, PLN, PRKAG2, TNNC1, TNNI3, TNNT2, TPM1*) were evaluated[15], and we compared findings with reference population datasets[9, 16-18], Caucasian HCM cohorts (n=6,179) [3, 19, 20] and performed functional studies using induced pluripotent stem cells derived cardiomyocytes (iPSC-CMs)[21, 22]. Detailed methods are available in the **Data Supplement**. All Singaporean participants gave written informed consent to participate in this ethics board approved study, which was carried out in accordance with local Tissue Acts. Supporting data are available either within the article and the Data Supplement or will be available on a reasonable request to the corresponding author due to privacy issue and national laws under the provision that data may not leave the hospital/center premises.

Results

The genetic architecture of HCM in Singapore

We sequenced 15 genes robustly associated with HCM and HCM pheno/genocopies in 224 unrelated, predominantly self-reported Chinese (78%) patients with a diagnosis of HCM. Preliminary annotations of variant pathogenicity were determined using CardioClassifier[15] with subsequent curation and validation by a clinical geneticist according to ACMG/AMP guidelines (Table S2, S3). The estimated contribution of each gene to disease was defined as the excess burden of rare protein-altering variants in cases compared with the population background (case excess), as previously described[19]. Population stratification assessment was performed using the genotype data of Singaporean HCM and Singaporean control cohorts and principal component analysis (PCA), which showed overlapping clusters of cases and controls (Figure S1).

American Heart Association.
Circulation: Genomic and Precision Medicine

/In Singaporean HCM patients, a significant case excess (p<0.0001) of variants was observed in the two thick filament genes (*MYBPC3*, 12.1%; *MYH7*, 9.1%) and the two main thin filament genes (*TNNI3*, 7.7%; *TNNT2*, 5.4%) (Table S4). There was insufficient statistical power to study other minor genes in detail, however the remaining sarcomeric genes (*ACTC1*, *MYL2*, *MYL3*, *TNNC1* and *TPM1*) had a combined case excess of 5.1%. Other HCM genes (*CSRP3*, *FHL1*, *PLN*) and pheno/genocopy genes (*GLA*, *LAMP2*, *PRKAG2*) exhibited few variants (2.0%) (Figure 2A and Table S4). Over 95% of the variant case excess (>92% of P/LP variants) were observed in the sarcomere-encoding genes. Overall, about 60% of Singaporean HCM patients had no disease-associated variant in the 15 core HCM genes. The mean left ventricular maximum wall thickness (LVMWT) in Singaporean HCM patients was 19.2mm as measured by cardiac magnetic resonance (CMR) or 20mm by echocardiogram (echo) imaging while the

average indexed LV mass (LVMi) was 92.3g/m^2 (CMR). There were no differences in cardiac phenotypes among P/LP variants in sarcomeric-positive carriers and non-carriers (Table S5).

We then compared the variant case excess frequency in the 15 HCM genes in Singaporeans patients with data from the Atlas of Cardiac Genetic Variation (ACGV) comprising up to 6,179 HCM cases, mostly (~75%) Caucasian[3, 19, 20]. Overall, the excess burden of rare variants (P/LP and VUS) in the 15 HCM-associated genes was similar in the two cohorts (41% in Singaporean, 38% in Caucasian) (Table 1). However, Singaporean patients had significantly fewer variants that could be interpreted with sufficient confidence for clinical decision making (P/LP) as compared to the Caucasian dataset (18% vs 31%, p<0.0001), but correspondingly had a much greater excess of VUS (exVUS: 24% vs 7%, p<0.0001) (Table 1). Interestingly, two thin filament genes associated with HCM, but with a modest prevalence of P/LP variants in Caucasians (Case Excess: *TNNI3*, 2.0%; *TNNT2*, 1.6%) had a far greater variant case excess in Singaporean HCM (*TNNI3*, 7.7%, p<0.0001; *TNNT2*, 5.4%, p=0.0005).

Circulation: Genomic and Precision Medicine

TNNI3:p.R79C and TNNT2:p.R286H variants are enriched in Singaporean HCM

We then studied *TNNI3* and *TNNT2* variants in more detail. While, there was no significant excess of P/LP variants in these genes (Table 1), two distinct VUS, *TNNI3*:p.R79C (ENST00000344887:c.235C>T) and *TNNT2*:p.R286H (ENST00000367318:c.857G>A) were commonly observed as heterozygous variant in 8/224 (3.6%) and 10/224 (4.5%) Singaporean HCM patients, respectively (Figure 2B). Carriers of these variants were self-reported Chinese and were mostly identified as Chinese using PCA analysis (Figure S2). Singaporean HCM carriers of *TNNI3*:p.R79C or *TNNT2*:p.R286H had similar cardiac morphology when compared to HCM patients with P or LP variants in these genes (Table S6).

The enrichment of the *TNNI3*:p.R79C and *TNNT2*:p.R286H variants in HCM cases compared to Caucasian patients was explored further using East Asian controls from gnomAD (gnomAD-EA) and also against 3,634 local Singaporean controls. *TNNT2*:p.R286H variants are rare (MAF<0.0001) while *TNNI3*:p.R79C has a MAF of 0.0004 in gnomAD (Table 2). The AF of *TNNI3*:p.R79C and *TNNT2*:p.R286H in gnomAD-EA or locally recruited Singaporean controls (in parentheses) was found to be higher: 0.0062 (0.0055) and 0.0009 (0.0017), respectively (Table 2). Similarly, *TNNI3*:p.R79C is common in Korean (gnomAD v2.1, AF=0.0063) and Japanese [Human Genetic Variation Database (HGVD), AF=0.0041] populations. *TNNT2*:p.R286H is not reported in either Korean or Japanese sub-continental population control datasets. Both variants are significantly enriched in HCM cases as compared to local Singaporean controls (*TNNI3*:p.R79C, p=0.0057 and *TNNT2*:p.R286H, p<0.0001) and when considered against gnomAD-EA (*TNNI3*:p.R79C, p=0.0086 and *TNNT2*:p.R286H, p<0.0001).

American Heart Association.
Circulation: Genomic and Precision Medicine

Attributable risk in cases with HCM

We then determined the effect of the two thin filament variants on HCM susceptibility. Using Singaporean controls as the population-specific reference, *TNNI3*:p.R79C has an odds ratio (OR (95% CI)) of 3.33 (1.54 – 7.20) and etiological fraction (EF (95% CI)) of 0.70 (0.35-0.86) (Table 3). The *TNNT2*:p.R286H variant has a higher OR (95% CI) of 14.1 (6.03 – 33.01) and EF (95% CI) of 0.93(0.83-0.97). Both *TNNI3*:p.R79C and *TNNT2*:p.R286H variants have been reported previously in HCM cases in China and Taiwan[23, 24] but with conflicting assessments of pathogenicity as shown in ClinVar (Table S7). *TNNI3*:p.R79C is classified mostly as likely benign while *TNNT2*:p.R286H is reported mostly as a VUS. Computational evidence supports a

potential pathogenic role as both variants are deleterious predictions by SIFT, Polyphen2 HumVar, MutationTaster and both have scaled CADD scores of >30 (top 0.1%) (Table S7).

Penetrance

Estimates of the population penetrance for *TNNI3*:p.R79C and *TNNT2*:p.R286H are both low (95% CI): 0.7% (0.2% - 1.7%) and 2.7% (0.8% - 8.6%) respectively (Table 3). These data are comparable with common HCM variants of low penetrance that have been reported in other populations[11]. To assess segregation and study further the penetrance of *TNNI3*:p.R79C and *TNNT2*:p.R286H, we invited all families of the genotype-positive individuals from HCM cases and volunteer controls, and ten families agreed to participate. The number of genotype positive individuals identified in family studies was small, but analyzing both variants together the aggregate penetrance was 22.2% (2.8% - 60.0%). Affected individuals in families studied were all >50, and many of the relatives assessed were younger (Figure S3). There were insufficient affected relatives to robustly assess segregation, though there were no phenotype positive individuals who did not carry the variant. Interestingly, in one family the younger proband was compound heterozygous for *TNNI3*:p.R79C and *TNNT2*:p.R286H and had more severe LVH (20mm) as compared to his elder sibling, who had the *TNNI3*:p.R79C variant only and relatively smaller LVMWT (15mm) despite the older age (Figure S3a).

American Heart Association.
Circulation: Genomic and Precision Medicine

Further family studies were conducted in Singaporean control subjects with *TNNI3*:p.R79C or *TNNT2*:p.R286H variants using cascade screening and CMR imaging. In Control Family 5, while the 30 years old proband had no HCM or LVH, the 57 year-old father who also has the *TNNI3*:p.R79C variant was diagnosed with HCM with patchy fibrosis during screening as part of this study (Figure S3b). The remaining three *TNNI3*:p.R79C families from the population controls and HCM cohort as well as the five *TNNT2*:p.R286H families from HCM

and controls cohorts who agreed to participate in this study were uninformative for segregation analyses (Figure S3c, d).

Expressivity of thin filament variants in the population

As measured by CMR, Singaporean population controls with *TNNI3*:p.R79C had significantly increased LVMi (52.1g/m^2, p=0.0219) and LVMWT (9.2mm, p=0.0001) when compared to the population controls without these variants (LVMi, 44.1g/m^2; LVMWT = 7.6mm) (Figure 3a, b; Table S8). Carriers of *TNNT2*:p.R286H had similar LVMWT (8.0mm, p>0.05) and LVMi (42.6g/m^2, p>0.05) when compared with non-carrier individuals from the general population.

Functional analyses of iPSC-CMs with TNNT2:p.R286H

To examine the functional effect of *TNNT2*:p.R286H, we generated isogenic iPSC-CMs with and without the heterozygous *TNNT2*:p.R286H (R286H/+) variant as well as a pathogenic HCM variant - *MYH7*:p.R403Q (R403Q/+) for a positive control, as previously described[25]. The contractile profiles, oxygen consumption rates (OCR), extracellular acidification rates (ECAR) and cell size were examined. Assessment of sarcomere function (Figure 4A, B) showed that R286H/+ and R403Q/+ iPSC-CMs had similar significantly increased contractility (p<0.0001) in comparison to the wildtype (WT). There was no significant change in the relaxation duration of R286H/+ iPSC-CMs while the R403Q/+ iPSC-CMs had significantly longer relaxation duration (p<0.0001) than R286H/+ iPSC-CMs and the WT. Significant increases in OCR (p=0.02) and ECAR (p=0.003) were observed in R286H/+ iPSC-CMs compared to the WT. However, both parameters were significantly more abnormal (Figure 4C, D) in R403Q/+ iPSC-CMs when compared to the R286H/+ iPSC-CMs (OCR, p<0.0001; ECAR, p=0.003) and WT (OCR and ECAR, p<0.0001).

Circulation: Genomic and Precision Medicine

Additionally, the mean area of the unconstrained WT, R403Q/+ and R286H/+ were compared to determine the cellular hypertrophy in comparison to the WT (Figure 4E). Note that each of the mutant cells are significantly larger than WT cells ($p<0.0001$) while the R286H/+ are also larger than p.R403Q/+ ($p<0.0001$). We suggest that the hypercontractility, increased metabolic requirements and cellular hypertrophy of the R286H/+ iPSC-CMs is consistent with cellular manifestations of HCM and therefore supports the likely pathogenicity of the *TNNT2:*p.R286H variant.

Haplotype analyses of TNNI3:p.R79C and TNNT2:p.R286H

To investigate the origins of these thin filament variants, we performed genome-wide genotyping of all carriers of *TNNI3*:p.R79C or *TNNT2*:p.R286H with or without HCM. We determined whether *TNNI3*:p.R79C and *TNNT2*:p.R286H are located on single haplotypes, suggesting a founder event, or multiple haplotypes suggestion more than one variant origin[26-28]. We observed *TNNI3*:p.R79C to be in linkage disequilibrium with two common single nucleotide polymorphisms (SNPs) (rs2288528 and rs2278281; Table S9 and Table S10) suggesting that *TNNI3*:p.R79C is a founder variant in Chinese.

American Heart Association.
Circulation: Genomic and Precision Medicine

Since all carriers of *TNNI3*:p.R79C and *TNNT2*:p.R286H in our study were of Chinese origin except one potentially a mixed descendent of Chinese and Malay, we further explored the AF of both variants in different Chinese provinces (*TNNI3*:p.R79C only) and other East Asian countries using data extracted from the CONVERGE study (n=11,670) (Table S11), the Taiwan biobank (n=1,517), the gnomAD-Korean (n=1,908) and the HGVD (Japanese, n=1,207) (Table 2). The AF of *TNNI3*:p.R79C in Fujian province located in Southeast China (0.0062), Taiwan (0.0060), South Korea (0.0063) and Japan (0.0041) were broadly similar to Singaporean (0.0055) yet relatively higher than other provinces in China (Figure S4). No common ancestral haplotype

block was found for *TNNT2*:p.R286H suggesting it is a recurrent variant. The prevalence of *TNNT2*:p.R286H in Taiwanese population (AF = 0.0013) is similar to Singaporean controls (AF = 0.0017) while the AF for *TNNT2*:p.R286H in Chinese provinces was not available (Figure S4).

Discussion

We used ACMG/AMP guidelines to identify disease variants for HCM in Singaporean patients. This revealed significantly fewer variants that could be robustly interpreted as P/LP and more VUSs in Singaporean HCM than reported in Caucasian HCM. These differences likely reflect the fact that Caucasian HCM has been very well studied and there are deep Caucasian control datasets, whereas non-Caucasian HCM is relatively unstudied. We surmise that the fewer P/LP variants seen in Singaporean HCM is due to the fact that while disease-causing variants exist, they have not been identified as such. This is in keeping with the increased levels of exVUS seen in Singaporean HCM.

The discoveries of founder and recurrent pathogenic variants with high levels of penetrance is recognized and reported[29-31]. However, incomplete penetrance occurs in HCM and current ACMG/AMP guidelines can inadequately classify variants. Low penetrance founder variants in *MYBPC3* are enriched in HCM cases in South Asian (c.3628-41_3628-17del25)[11] and Icelandic (c.927-2A>G) populations[12]. However, low penetrance HCM related variants have not been reported previously in thin filament sarcomeric genes and *TNNI3*:p.R79C and *TNNT2*:p.R286H, which we describe here, are the first such reported.

Although there were a limited number of families for segregation studies, the observed aggregated penetrance in families (22.2%) was higher than the predicted combined population penetrance at 3.4% [0.7% (TNNI3:p.R79C); 2.7% (TNNT2:p.R286H)]. However, lifestyle,

Circulation: Genomic and Precision Medicine

environmental factors[32] or modifier genes[33] among family members can also contribute to apparent measures of variant penetrance in the families. Of note, during this study we observed five Caucasian HCM cases with *TNNT2*:p.R278C from a UK HCM cohort (Table 3) with low penetrance (*TNNT2*:p.R278C, 0.012, 95% CI: 0.004-0.035) similar to the Chinese variants in *TNNT2* and *TNNI3* described here.

While the penetrance of the thin filament variants we describe here is low, they could account for a meaningful proportion of disease risk in patients with HCM who are found to carry these variants (EF of 0.93 (95% CI: 0.83-0.97) for *TNNT2:*R286H, (EF of 0.70 (95% CI: 0.35-0.86) for *TNNI3:*p.R79C). Using current ACMG/AMP guideline and data reported here, the *TNNT2*:p.R286H variant could be reconsidered as LP instead of VUS when rule BS1 (the presence of the variant in Singaporean population controls) is revised with rule PS4 (significantly higher prevalence of the variant in Singaporean HCM patients). The deleterious effects of *TNNT2*:p.R286H seen in the functional studies (rule PS3) further support a possible reclassification in Singapore. Meanwhile, *TNNI3:*p.R79C variant might be reclassified as VUS from Benign/Likely Benign (8 studies in ClinVar, Table S7) when rule BS1 and PP3 are activated.

American Heart Association.
Circulation: Genomic and Precision Medicine

Our functional studies of iPSC-CMs encoding *TNNT2*:p.R286H showed hypercontractility, cellular hypertrophy, and higher metabolic requirements than isogenic WT iPSC-CMs. These parameters are also abnormal in isogenic iPSC-CMs encoding the definitive HCM pathogenic variant *MYH7*:p.R403Q, supporting our conclusion that *TNNT2*:p.R286H is a likely pathogenic variant. However, we note that the *TNNT2*:p.R286H variant did not perturb relaxation times, unlike the *MYH7*:p.R403Q variant and that all of the parameters studied were significantly more abnormal in *MYH7*:p.R403Q than in *TNNT2*:p.R286H. These differences

may imply distinct mechanisms by which thick and thin filament pathogenic variants cause HCM. For example, abnormal sarcomere function might occur from thick filament variants by their influence on the conformational states of myosin[25], while thin filament variants may alter sarcomere function by influencing calcium sensitivity. Irrespective of this or other mechanisms that account for these differences, we suggest that normal relaxation and attenuated dysfunction of other parameters that we observed in TNNT2:R286H iPSC-CMs and could account for milder phenotype and reduced disease penetrance among HCM patients with this variant.

A limitation of our study is that we did not functionally validate the TNNI3.pR79C variant, which will be reported in follow-on studies. We noted elevated left ventricular mass and wall thickness in the general population with the TNNI3.pR79C variant, which requires further study. Given the size of the Chinese population, the *TNNT2* and *TNNI3* variants described here could be associated with a theoretical 200,000 cases of HCM in China. Further work is needed to determine the implications of our findings given the penetrance of both variants is low for overt HCM and replication and extension studies are required to assign robustness for clinical interpretation.

Circulation: Genomic and Precision Medicine

Acknowledgments: We would like to thank Professor Jonathan Flint for sharing the data of CONVERGE study, all contributors to the SingHealth Exome Consortium (SEC), the healthy volunteers, patients and families for participating in this study. We also thank clinical research coordinators for recruiting patients and families.

Sources of Funding: The research was supported in part by the National Medical Research Council (NMRC) Singapore STaR awards (NMRC/STaR/0011/2012 and NMRC/STaR/0029/2017 to S.A.C), Goh Foundation, and Tanoto Foundation, the NMRC Centre Grant and Collaborative Centre Grant schemes (NMRC/CGAug16C006 to the NHCS), NHCS Centre Grant Seed Funding (NHCS-CGSF/2018/001 to P.C.J), NIHR Imperial College

Biomedical Research Centre, Wellcome Trust (107469/Z/15/Z to J.S.W.), Wellcome Trust Sir Henry Wellcome fellowship (206466/Z/17/Z to C.N.T), Medical Research Council (intramural awards to S.A.C. and J.S.W), Health Innovation Challenge Fund award from the Wellcome Trust and Department of Health (UK; HICF-R6-373; S.A.C., J.S. W.), the British Heart Foundation (SP/10/10/28431 to S.A.C), BHF Centre of Research excellence Intermediate Transition Fellowship (C.N.T), Sarnoff Foundation (A.C.G, G.G.R), Fondation Leducq (S.A.C., C.E.S., J.G.S.), São Paulo Research Foundation (FAPESP 2019/11821-1 to G.V.). The views expressed in this work are those of the authors, and the funding institutions played no role in the design, collection, analysis, or interpretation of the data or in the decision to submit the manuscript for publication.

Disclosures: None.

American Heart Association.

References:

1. Maron BJ. Hypertrophic cardiomyopathy: a systematic review. *JAMA*. 2002;287:1308-20.

2. Elliott PM, Anastasakis A, Borger MA, Borggrefe M, Cecchi F, Charron P, Hagege AA, Lafont A, Limongelli G, Mahrholdt H, *et al.* 2014 ESC Guidelines on diagnosis and management of hypertrophic cardiomyopathy: the Task Force for the Diagnosis and Management of Hypertrophic Cardiomyopathy of the European Society of Cardiology (ESC). *Eur Heart J*. 2014;35:2733-79.

Circulation: Genomic and Precision Medicine

3. Walsh R, Buchan R, Wilk A, John S, Felkin LE, Thomson KL, Chiaw TH, Loong CCW, Pua CJ, Raphael C, *et al.* Defining the genetic architecture of hypertrophic cardiomyopathy: re-evaluating the role of non-sarcomeric genes. *Eur Heart J*. 2017;38:3461-3468.

4. Jarcho JA, McKenna W, Pare JA, Solomon SD, Holcombe RF, Dickie S, Levi T, Donis-Keller H, Seidman JG and Seidman CE. Mapping a gene for familial hypertrophic cardiomyopathy to chromosome 14q1. *N Engl J Med*. 1989;321:1372-8.

5. Rehm HL, Berg JS, Brooks LD, Bustamante CD, Evans JP, Landrum MJ, Ledbetter DH, Maglott DR, Martin CL, Nussbaum RL, *et al.* ClinGen--the Clinical Genome Resource. *N Engl J Med*. 2015;372:2235-42.

6. Ingles J, Goldstein J, Thaxton C, Caleshu C, Corty EW, Crowley SB, Dougherty K, Harrison SM, McGlaughon J, Milko LV, *et al.* Evaluating the Clinical Validity of Hypertrophic Cardiomyopathy Genes. *Circ Genom Precis Med*. 2019;12:e002460.

7. Richards S, Aziz N, Bale S, Bick D, Das S, Gastier-Foster J, Grody WW, Hegde M, Lyon E, Spector E, *et al.* Standards and guidelines for the interpretation of sequence variants: a joint consensus recommendation of the American College of Medical Genetics and Genomics and the Association for Molecular Pathology. *Genet Med*. 2015;17:405-24.

8. Whiffin N, Minikel E, Walsh R, O'Donnell-Luria AH, Karczewski K, Ing AY, Barton PJR, Funke B, Cook SA, MacArthur D, *et al.* Using high-resolution variant frequencies to empower clinical genome interpretation. *Genet Med*. 2017;19:1151-1158.

9. Karczewski KJ, Francioli LC, Tiao G, Cummings BB, Alföldi J, Wang Q, Collins RL, Laricchia KM, Ganna A, Birnbaum DP, *et al.* Variation across 141,456 human exomes and genomes reveals the spectrum of loss-of-function intolerance across human protein-coding genes. *bioRxiv*. 2019:531210.

10. Ross SB, Bagnall RD, Ingles J, Van Tintelen JP and Semsarian C. Burden of Recurrent and Ancestral Mutations in Families With Hypertrophic Cardiomyopathy. *Circ Cardiovasc Genet*. 2017;10.

11. Dhandapany PS, Sadayappan S, Xue Y, Powell GT, Rani DS, Nallari P, Rai TS, Khullar M, Soares P, Bahl A, *et al.* A common MYBPC3 (cardiac myosin binding protein C) variant associated with cardiomyopathies in South Asia. *Nat Genet*. 2009;41:187-91.

12. Adalsteinsdottir B, Teekakirikul P, Maron BJ, Burke MA, Gudbjartsson DF, Holm H, Stefansson K, DePalma SR, Mazaika E, McDonough B, *et al.* Nationwide study on hypertrophic cardiomyopathy in Iceland: evidence of a MYBPC3 founder mutation. *Circulation*. 2014;130:1158-67.

13. Bylstra Y, Kuan JL, Lim WK, Bhalshankar JD, Teo JX, Davila S, Teh BT, Rozen S, Tan EC, Liew WKM, *et al.* Population genomics in South East Asia captures unexpectedly high carrier frequency for treatable inherited disorders. *Genet Med*. 2019;21:207-212.

14. Pua CJ, Bhalshankar J, Miao K, Walsh R, John S, Lim SQ, Chow K, Buchan R, Soh BY, Lio PM, *et al.* Development of a Comprehensive Sequencing Assay for Inherited Cardiac Condition Genes. *J Cardiovasc Transl Res*. 2016;9:3-11.

15. Whiffin N, Walsh R, Govind R, Edwards M, Ahmad M, Zhang X, Tayal U, Buchan R, Midwinter W, Wilk AE, *et al.* CardioClassifier: disease- and gene-specific computational decision support for clinical genome interpretation. *Genet Med*. 2018.

16. Chen CH, Yang JH, Chiang CWK, Hsiung CN, Wu PE, Chang LC, Chu HW, Chang J, Song IW, Yang SL, *et al.* Population structure of Han Chinese in the modern Taiwanese population based on 10,000 participants in the Taiwan Biobank project. *Hum Mol Genet*. 2016;25:5321-5331.

American Heart Association.
Circulation: Genomic and Precision Medicine

17. Higasa K, Miyake N, Yoshimura J, Okamura K, Niihori T, Saitsu H, Doi K, Shimizu M, Nakabayashi K, Aoki Y, *et al.* Human genetic variation database, a reference database of genetic variations in the Japanese population. *J Hum Genet*. 2016;61:547-53.

18. Cai N, Bigdeli TB, Kretzschmar WW, Li Y, Liang J, Hu J, Peterson RE, Bacanu S, Webb BT, Riley B, *et al.* 11,670 whole-genome sequences representative of the Han Chinese population from the CONVERGE project. *Sci Data*. 2017;4:170011.

19. Walsh R, Thomson KL, Ware JS, Funke BH, Woodley J, McGuire KJ, Mazzarotto F, Blair E, Seller A, Taylor JC, *et al.* Reassessment of Mendelian gene pathogenicity using 7,855 cardiomyopathy cases and 60,706 reference samples. *Genet Med*. 2017;19:192-203.

20. Alfares AA, Kelly MA, McDermott G, Funke BH, Lebo MS, Baxter SB, Shen J, McLaughlin HM, Clark EH, Babb LJ, *et al.* Results of clinical genetic testing of 2,912 probands with hypertrophic cardiomyopathy: expanded panels offer limited additional sensitivity. *Genet Med*. 2015;17:880-8.

21. Sharma A, Toepfer CN, Schmid M, Garfinkel AC and Seidman CE. Differentiation and Contractile Analysis of GFP-Sarcomere Reporter hiPSC-Cardiomyocytes. *Curr Protoc Hum Genet*. 2018;96:21.12.1-21.12.12.

22. Toepfer CN, Sharma A, Cicconet M, Garfinkel AC, Mucke M, Neyazi M, Willcox JAL, Agarwal R, Schmid M, Rao J, *et al.* SarcTrack. *Circ Res*. 2019;124:1172-1183.

23. Chiou KR, Chu CT and Charng MJ. Detection of mutations in symptomatic patients with hypertrophic cardiomyopathy in Taiwan. *J Cardiol*. 2015;65:250-6.

24. Tao Q, Yang J, Cheng W, Yu S, Fang X, He P and Zhang Y. A novel TNNI3 gene mutation (c.235C>T/ p.Arg79Cys) found in a thirty-eight-year-old women with hypertrophic cardiomyopathy. *Open Life Sciences*. 2018;13:374.

25. Toepfer CN, Garfinkel AC, Venturini G, Wakimoto H, Repetti G, Alamo L, Sharma A, Agarwal R, Ewoldt JF, Cloonan P, *et al.* Myosin Sequestration Regulates Sarcomere Function, Cardiomyocyte Energetics, and Metabolism, Informing the Pathogenesis of Hypertrophic Cardiomyopathy. *Circulation*. 2020.

26. Raychaudhuri S, Iartchouk O, Chin K, Tan PL, Tai AK, Ripke S, Gowrisankar S, Vemuri S, Montgomery K, Yu Y, *et al.* A rare penetrant mutation in CFH confers high risk of age-related macular degeneration. *Nat Genet*. 2011;43:1232-6.

27. Aung T, Ozaki M, Lee MC, Schlotzer-Schrehardt U, Thorleifsson G, Mizoguchi T, Igo RP, Jr., Haripriya A, Williams SE, Astakhov YS, *et al.* Genetic association study of exfoliation syndrome identifies a protective rare variant at LOXL1 and five new susceptibility loci. *Nat Genet*. 2017;49:993-1004.

American Heart Association.
Circulation: Genomic and Precision Medicine

28. Jin SC, Homsy J, Zaidi S, Lu Q, Morton S, DePalma SR, Zeng X, Qi H, Chang W, Sierant MC, *et al.* Contribution of rare inherited and de novo variants in 2,871 congenital heart disease probands. *Nat Genet*. 2017;49:1593-1601.

29. Alders M, Jongbloed R, Deelen W, van den Wijngaard A, Doevendans P, Ten Cate F, Regitz-Zagrosek V, Vosberg HP, van Langen I, Wilde A, *et al.* The 2373insG mutation in the MYBPC3 gene is a founder mutation, which accounts for nearly one-fourth of the HCM cases in the Netherlands. *Eur Heart J*. 2003;24:1848-53.

30. Jaaskelainen P, Helio T, Aalto-Setala K, Kaartinen M, Ilveskoski E, Hamalainen L, Melin J, Nieminen MS, Laakso M, Kuusisto J, *et al.* Two founder mutations in the alpha-tropomyosin and the cardiac myosin-binding protein C genes are common causes of hypertrophic cardiomyopathy in the Finnish population. *Ann Med*. 2013;45:85-90.

31. Sabater-Molina M, Saura D, Garcia-Molina Saez E, Gonzalez-Carrillo J, Polo L, Perez-Sanchez I, Olmo MDC, Oliva-Sandoval MJ, Barriales-Villa R, Carbonell P, *et al.* A Novel Founder Mutation in MYBPC3: Phenotypic Comparison With the Most Prevalent MYBPC3 Mutation in Spain. *Rev Esp Cardiol (Engl Ed)*. 2017;70:105-114.

32. Pasipoularides A. Genomic translational research: Paving the way to individualized cardiac functional analyses and personalized cardiology. *Int J Cardiol*. 2017;230:384-401.

33. Daw EW, Chen SN, Czernuszewicz G, Lombardi R, Lu Y, Ma J, Roberts R, Shete S and Marian AJ. Genome-wide mapping of modifier chromosomal loci for human hypertrophic cardiomyopathy. *Hum Mol Genet*. 2007;16:2463-71.

Circulation: Genomic and Precision Medicine

Table 1. Frequencies of HCM variant case excesses in fifteen HCM genes in Singaporean and Caucasian patients

Gene	P/LP (%)			exVUS (%)			Case Excess (P/LP/exVUS) (%)		
	Singaporean (n = 224)	UK/US (ACGV) (n= 632-6,179)	Fisher's exact p-value	Singaporean (n = 224)	UK/US (ACGV) (n= 632-6,179)	Fisher's exact p-value	Singaporean (n = 224)	UK/US (ACGV) (n= 632-6,179)	Fisher's exact p-value
**Sarcomeric genes**									
MYBPC3	16 (7.1)	924 (15.0)	**0.0007**	11 (5.0)	90 (1.5)	**0.0007**	27 (12.1)	1014 (16.4)	0.0964
MYH7	10 (4.5)	608 (9.9)	0.0040	10 (4.6)	134 (2.2)	0.0362	20 (9.1)	742 (12.1)	0.1732
TNNI3	7 (3.1)	89 (1.5)	0.0848	10 (4.6)	30 (0.5)	**<0.0001**	17 (7.7)	119 (2.0)	**<0.0001**
TNNT2	3 (1.3)	73 (1.2)	0.7511	9 (4.1)	25 (0.4)	**<0.0001**	12 (5.4)	98 (1.6)	**0.0005**
TPM1	0 (0.0)	31 (0.7)	0.4009	5 (2.1)	29 (0.7)	0.0216	4 (2.1)	60 (1.3)	0.5485
TNNC1	0 (0.0)	0 (0.0)	1.0000	4 (1.7)	no excess	0.0046	4 (1.7)	no excess	0.0046
ACTC1	1 (0.4)	7 (0.2)	0.3413	1 (0.4)	11 (0.3)	0.4655	2 (0.9)	18 (0.4)	0.2702
MYL2	0 (0.0)	25 (0.6)	0.6358	1 (0.3)	11 (0.3)	0.4655	1 (0.3)	38 (0.9)	0.7209
MYL3	0 (0.0)	9 (0.2)	1.0000	0 (0.1)	11 (0.3)	1.0000	0 (0.1)	20 (0.5)	0.6219
**Other HCM genes**									
FHL1	1 (0.4)	1 (0.1)	0.2385	1 (0.3)	10 (0.6)	1.0000	2 (0.8)	11 (0.7)	1.0000
CSRP3	0 (0.0)	3 (0.1)	1.0000	0 (0.1)	no excess	1.0000	0 (0.1)	3 (0.1)	1.0000
PLN	0 (0.0)	2 (0.1)	1.0000	no excess	5 (0.2)	1.0000	no excess	7 (0.3)	1.0000
**Pheno/Genocopy genes**									
GLA	2 (0.9)	21 (0.6)	0.3812	1 (0.3)	no excess	0.0571	3 (1.2)	19 (0.5)	0.3391
LAMP2	0 (0.0)	16 (0.5)	0.6194	no excess	no excess	1.0000	no excess	10 (0.3)	1.0000
PRKAG2	0 (0.0)	12 (0.3)	1.0000	no excess	3 (0.1)	1.0000	no excess	15 (0.4)	1.0000
Total	40 (17.9)	1915 (31.0)*	**<0.0001**	53 (23.6)	439 (7.1)*	**<0.0001**	92 (41.2)	2317 (37.5)*	0.2329

ACGV = Atlas of Cardiac Genetic Variation[3,13,14]; P, pathogenic; LP, likely pathogenic; exVUS, excess in VUS; n/s, not significant; *, Total cases are derived using the total percentage of positive cases multiplied by the maximum total number of cases in MYBPC3 (n=6,179) for Fisher's exact test; Fisher's exact p-value in **bold** (<0.0033) indicates a significant excess, corrected for multiple testing (n=15)

Circulation: Genomic and Precision Medicine

Table 2. Allele frequencies of *TNNI3*:p.R79C (rs3729712) or *TNNT2*:p.R286H (rs141121678) in Singaporean HCM cases and controls and in different population controls

Cohorts	Study Populations		*TNNI3*:p.R79C			*TNNT2*:p.R286H		
			Allele Count	Allele Number	Allele Frequency	Allele Count	Allele Number	Allele Frequency
**HCM**								
	**Singaporean**		8	448	0.01786	10	448	0.02232
**Control**								
	**Singaporean**		40	7268	0.00550	12	7268	0.00165
	**gnomAD**							
	East Asian	Other East Asian	95	15414	0.00616	17	15594	0.00109
		Korean	24	3816	0.00629	0	3814	0
		Japanese	1	82	0.01220	0	134	0
		Overall	120	19312	0.00621	17	19542	0.00087
	South Asian		5	30326	0.00016	0	28848	0
	European (Non-Finnish)		0	123074	0	2	125278	0.00002
	European (Finnish)		0	24786	0	0	24260	0
	African		0	22552	0	0	24214	0
	Ashkenazi Jewish		0	10134	0	0	10122	0
	Latino		1	35024	0.00003	0	34676	0
	Other		0	6980	0	0	6996	0
	**Taiwan Biobank**							
	Taiwanese		18	3000	0.00600	4	3030	0.00132
	**The Human Genetic Variation Database (HGVD)**							
	Japanese		10	2414	0.00414	0	2414	0

American Heart Association.
Circulation: Genomic and Precision Medicine

Table 3. Comparison of Singaporean *TNNI3* and *TNNT2* variants with other common HCM variants of reduced penetrance

Gene	Coding HGVS	Protein HGVS	Country	HCM Prevalence	Control Prevalence	Fisher's exact p-value	OR (95% CI)	EF (95% CI)	Penetrance (95% CI)	Clin Var	Pubmed ID
MYBPC3	c.927-2A>G	-	Iceland	88/151 (58.3%)	355/98721 (0.4%)*	**<0.0001**	387.04 (275.53-543.69)	1 (1.00-1.00)	0.32 (0.24-0.43)	P	25078086
MYBPC3	c.3628-41_3628-17del25	-	India	87/800 (10.9%)	962/15296 (6.3%)†	**<0.0001**	1.81 (1.44-2.29)	0.45 (0.31-0.56)	0.004 (0.003-0.005)	C	19151713
TNNI3	c.235C>T	p.R79C	Singapore	8/224 (3.6%)	40/3634 (1.1%)‡	**0.0057**	3.33 (1.54-7.20)	0.70 (0.35-0.86)	0.007 (0.002-0.017)	C	-
TNNT2	c.857G>A	p.R286H	Singapore	10/224 (4.4%)	12/3634 (0.3%)‡	**<0.0001**	14.1 (6.03-33.01)	0.93 (0.83-0.97)	0.027 (0.008-0.086)	C	-
TNNT2	c.832C>T	p.R278C	UK	5/685 (0.7%)	76/62835 (0.1%)§	**<0.0001**	6.07 (2.45-15.06)	0.84 (0.59-0.93)	0.012 (0.004-0.035)	C	-

*, deCODE genomic genealogical data; †, gnomAD (South Asian); ‡, Singaporean Controls; §, gnomAD (European Non-Finnish); OR, Odd ratio; EF, Etiological fraction; P, pathogenic; LP, likely pathogenic; C, conflicting submission; Fisher's exact p-value in **bold** indicates a significant excess

Circulation: Genomic and Precision Medicine

Figure Legends:

Figure 1. Overview of the study design outlining the major components of the study and the various disease and control populations used. ACGV, Atlas of Cardiac Genetic Variation[3,13,14]; AF, allele frequency; SG, Singaporean; P, pathogenic, LP, likely pathogenic; exVUS, excess variant of unknown significance; EA, East Asian; *, https://www.cardioclassifier.org/[15]

Figure 2. Pathogenic/likely pathogenic variants and excess VUS (exVUS = caseVUS%-controlVUS%) in HCM genes in Singaporean HCM patients. **A)** Fifteen genes were assessed including major sarcomeric genes (*MYBPC3*, *MYH7*, *TNNT2*, *TNNI3*), other sarcomeric genes (*ACTC*, *MYL2*, *MYL3*, *TPM1*, *TNNC1*), other HCM genes (*CSRP3*, *FHL1*, *PLN*) and geno/phenocopies (*GLA*, *LAMP*, *PRKAG2*). The number and percentage refer to the total P/LP case per gene (darker shade) while the number and percentage in parentheses refer to the total case excess of P, LP and exVUS (lighter shade) as compared to gnomAD. **B)** The secondary pie charts show the proportion of all Singaporean HCM patients with *TNNI3*:p.R79C or *TNNT2*:p.R286H VUS as compared to other VUS in these genes, depicted overall in (a) by lighter shading.

Circulation: Genomic and Precision Medicine

Figure 3. *TNNI3*:p.R79C and *TNNT2*:p.R286H: Cardiac indices in the general population. Violin plots comparing **A)** LVMi and **B)** LVMWT in population controls with or without *TNNI3*:p.R79C or *TNNT2*:p.R286H, derived using CMR. Data were represented as median ± interquartile range (IQR) in a violin box-and-whiskers plot (Tukey's rule) with the whiskers

representing 1.5x IQR and outliers were plotted as individual dots. *P* values of regression models was derived using ANOVA where a significance cut-off of $p<0.05$ was used.

Figure 4. Contractile characterization, metabolic flux and cell size analysis of *TNNT2:*p.R286H iPSC-CMs. **A)** Comparison of the percentage sarcomere shortening and **B)** relaxation duration for isogenic wildtype (WT), *TNNT2*:p.R286H (R286H/+) and established (+) hypertrophic variant of *MYH7*:p.R403Q (R403Q/+) iPSC-CMs. **C)** Measurement of oxygen consumption rate (OCR) and **D)** extracellular acidification rate (ECAR) in WT, R286H/+ and R403Q/+ cardiomyocytes using the Seahorse platform and **E)** unconstrained cell size in WT (n = 586 cells), R286H/+ (n = 408 cells) and (+) R403Q/+ (n = 488 cells). All iPSC-CMs were generated by mutating an isogenic line, denoted TTN-GFP PGP1[21,22]. Two or more differentiations were studied from two independent clones for each genotype. Data, mean ± SEM. Student's t-test for each mutant compared to WT was used where a significance cut-off of $p<0.05$ was used.

Circulation: Genomic and Precision Medicine

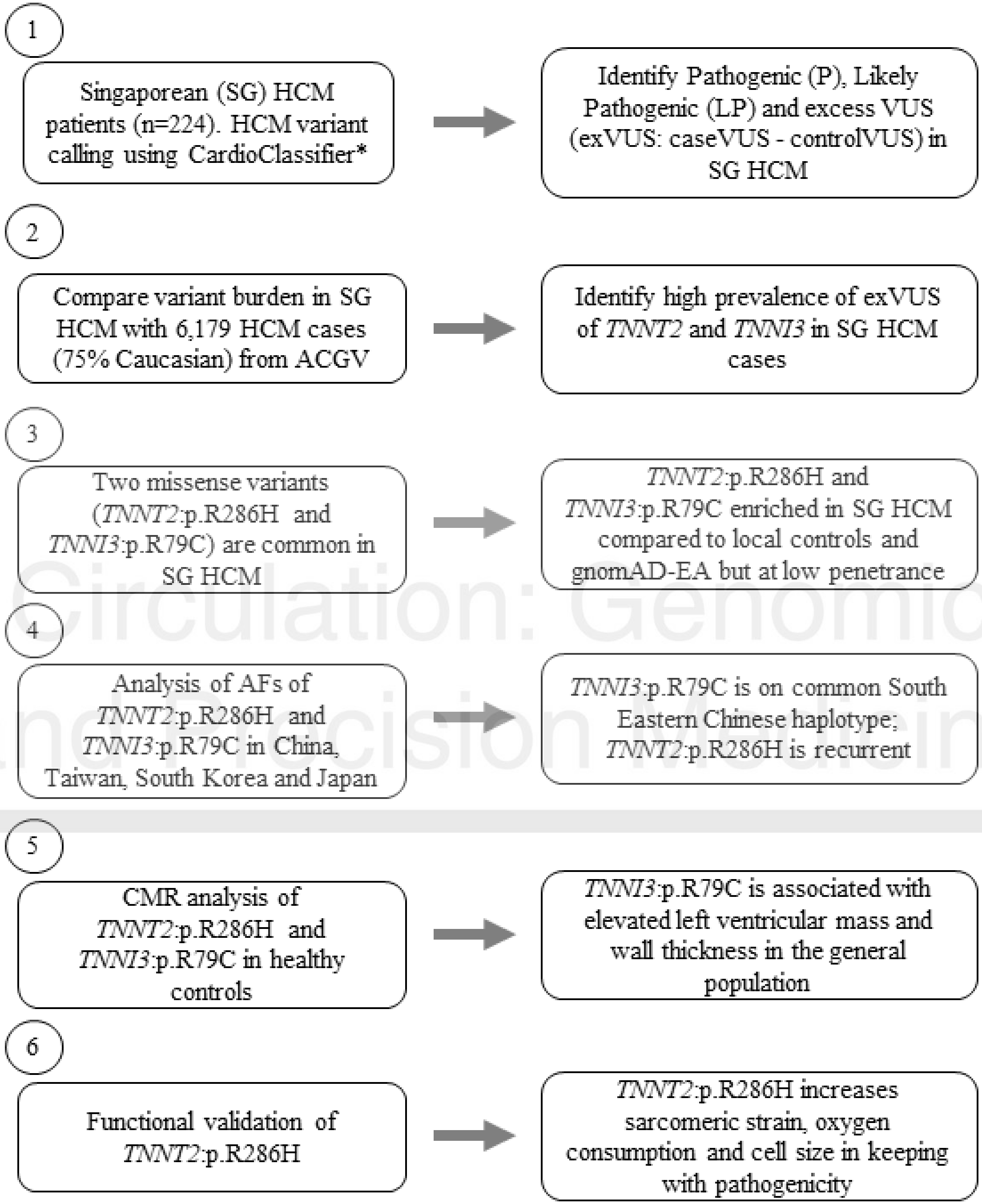

American Heart Association.
Circulation: Genomic and Precision Medicine

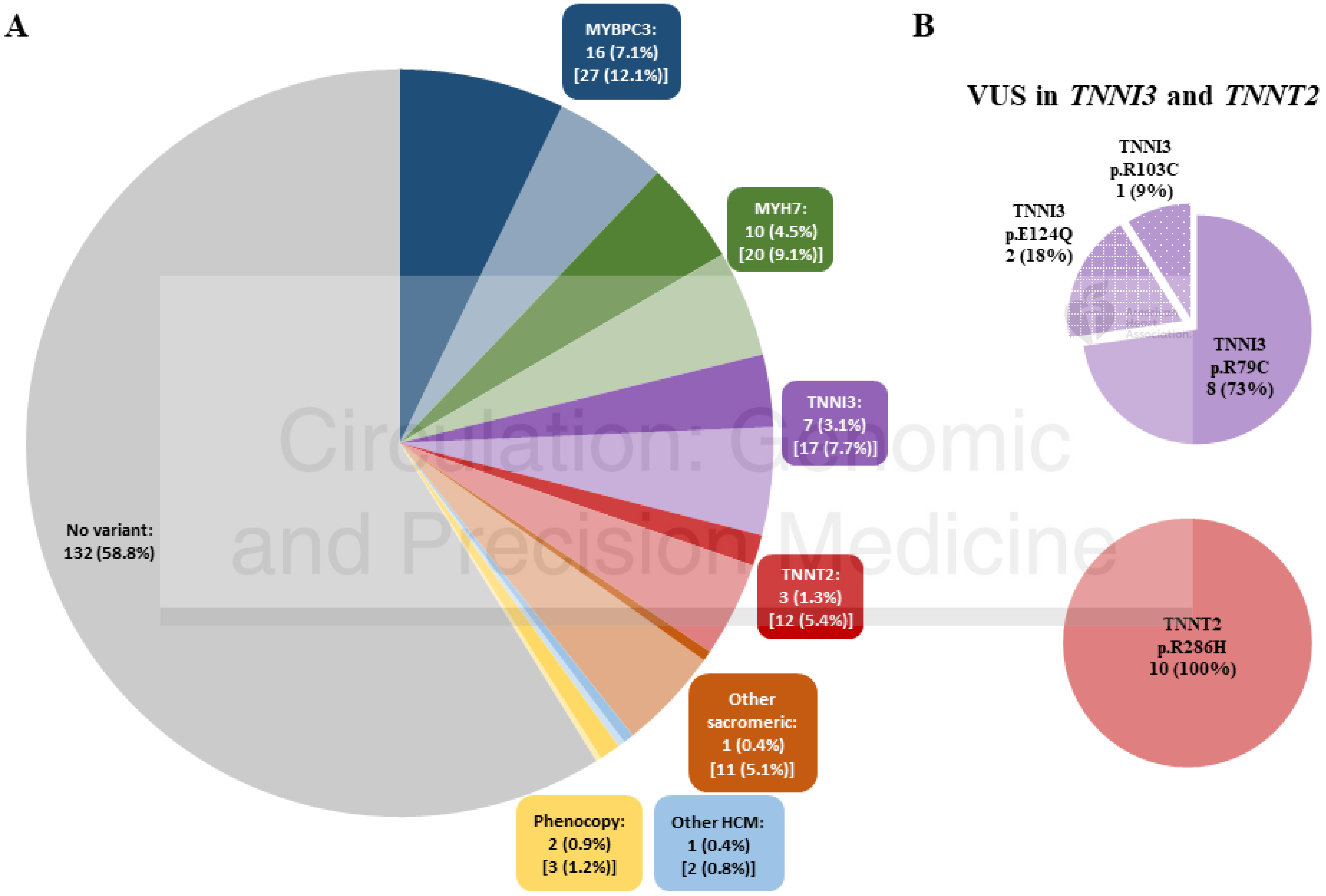

Circulation: Genomic and Precision Medicine

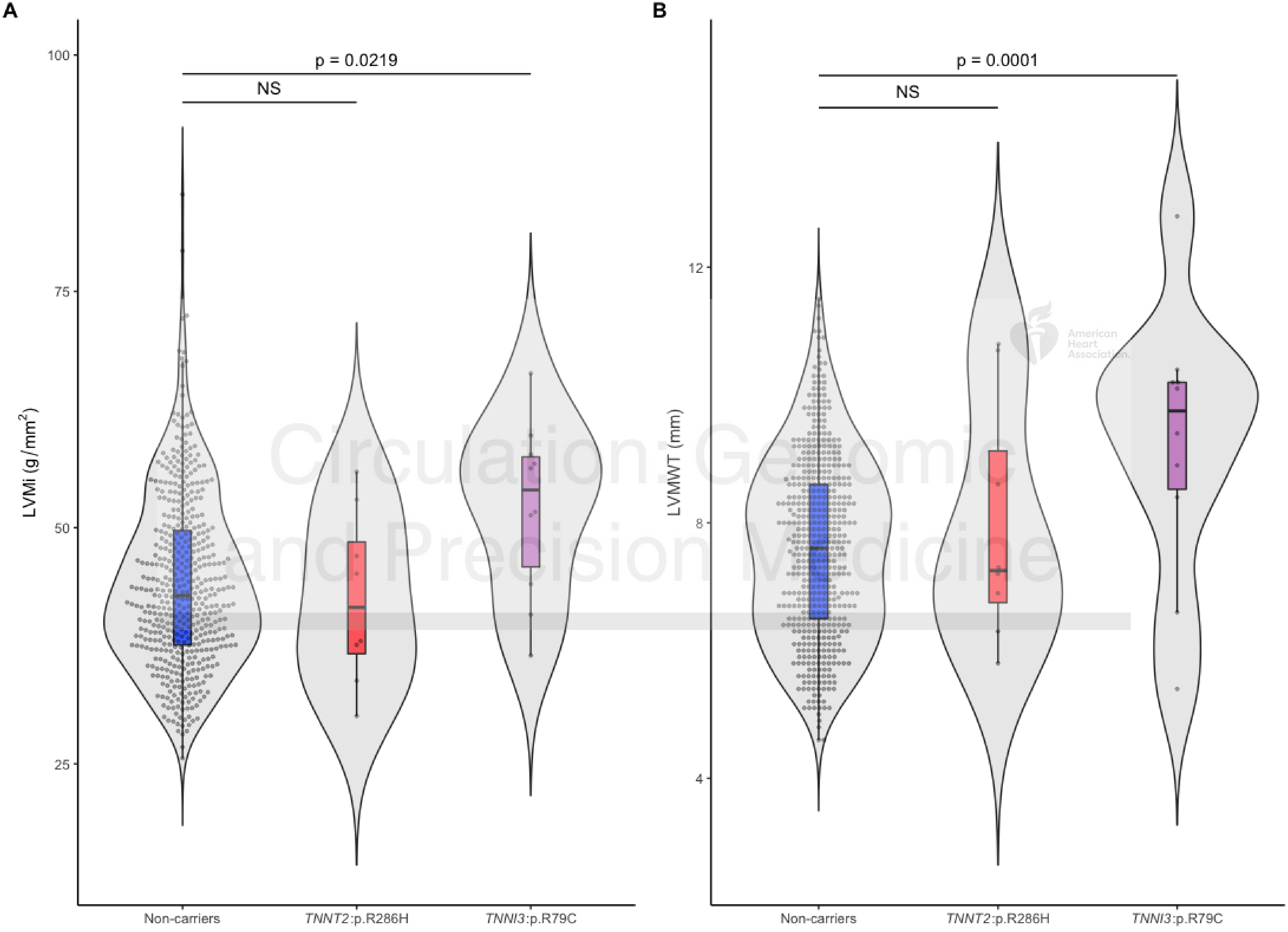
A
B
p = 0.0219
NS
p = 0.0001
NS
LVMi (g/mm^2)
LVMWT (mm)
100
75
50
25
12
8
4
Non-carriers
TNNT2:p.R286H
TNNI3:p.R79C
Non-carriers
TNNT2:p.R286H
TNNI3:p.R79C

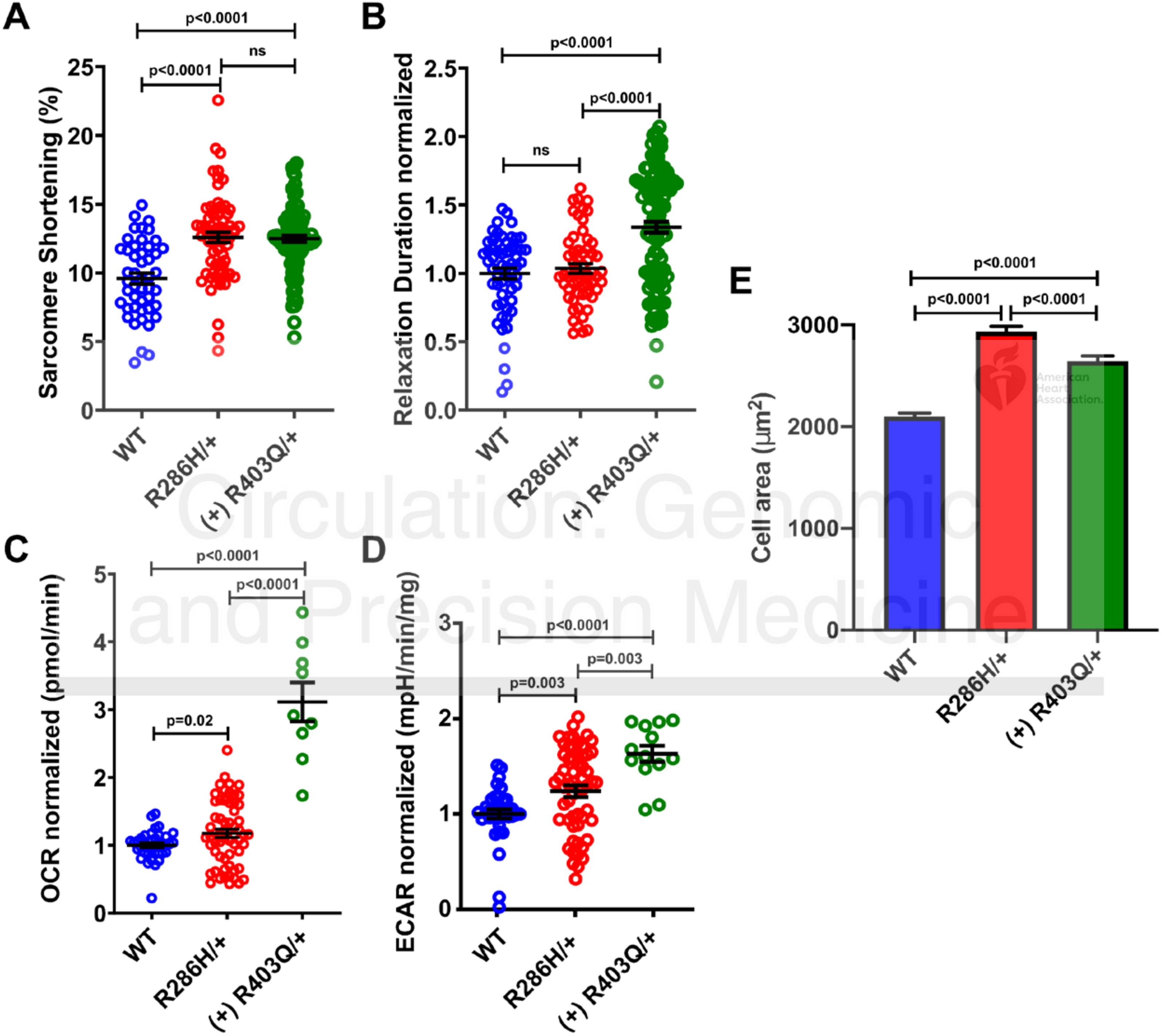

Circulation: Genomic and Precision Medicine
